# The overall and smoking-attributable burden of multiple sclerosis among older adults aged 65–89 years from 1990 to 2019 and predictions to 2040

**DOI:** 10.3389/fmed.2024.1430741

**Published:** 2024-08-22

**Authors:** Jiao Su, Yuanhao Liang, Xiaofeng He

**Affiliations:** ^1^Department of Biochemistry, Changzhi Medical College, Changzhi, China; ^2^Clinical Experimental Center, Jiangmen Key Laboratory of Clinical Biobanks and Translational Research, Jiangmen Central Hospital, Jiangmen, China; ^3^Institute of Evidence-Based Medicine, Heping Hospital Affiliated to Changzhi Medical College, Changzhi, China

**Keywords:** multiple sclerosis, global burden of disease, age-standardized rate, estimated annual percentage change, older adults, smoking

## Abstract

**Background:**

The global prevalence of aging individuals with multiple sclerosis (MS) is increasing. This study aimed to assess the burden and trends of overall and smoking-attributable MS in older adults aged 65–89 years at the global, regional, and national levels.

**Methods:**

The number and rates of years of life lived with disability (YLD) and years of life lost (YLL) due to MS for older adults in 204 countries and territories from 1990 to 2019 were retrieved from the Global Burden of Disease (GBD) Study 2019. Estimated annual percentage change (EAPC) in the age-standardized YLD and YLL rates were calculated to quantify the temporal trends. The Bayesian age-period-cohort model was used to predict the trends from 2020 to 2040.

**Results:**

In 2019, there were an estimated 80,040 (95% uncertainty interval 57,534 to 103,608) YLD and 139,132 (107,632 to 161,172) YLL caused by MS among older adults globally. The age-standardized YLD and YLL rates decreased by an average of −0.21% (95% CI –0.26 to −0.16) and − 0.2% (95% CI –0.26 to −0.14) per year for overall MS from 1990 to 2019, respectively. The number of YLL globally in 2019 was 7,891 (5,003 to 10,991) and 15,667 (10,833 to 20,076) due to smoking-attributable MS. The age-standardized YLD and YLL rates decreased by an annual average of −1.14% (95% CI –1.25 to −1.04) and − 1.15% (95% CI –1.27 to −1.03) for MS attributable to smoking. Although the global age-standardized rates of YLD and YLL for MS among older adults declined from 1990 to 2019, many regions showed increases. The largest increase in age-standardized YLD rate of MS was observed in East Asia (average annual change 1.62% [95% CI: 1.56 to 1.68]), while the largest increase in the age-standardized YLL rate occurred in High-income North America (1.74% [1.53 to 1.96]). Nationally, the age-standardized YLD and YLL rates for overall and smoking-attributable MS increased exponentially with increases in SDI level (all model *p* < 0.001). Furthermore, projections have also indicated an expected decrease in the age-standardized rates of YLD and YLL of MS in the elderly population from 2020 to 2040.

**Conclusion:**

Tracking trends in MS burden among older adults provides insights into the potential shifts in disease patterns over time. The findings lay the groundwork for informed decision-making in public health and healthcare delivery, aiming to ensure that older adults with MS receive appropriate care and support.

## 1 Introduction

Multiple sclerosis (MS) is an inflammatory demyelinating and neurodegenerative disorder of the human central nervous system, which can cause a wide range of symptoms over the course of disease and its severity vary depending on the location of affected nerve fibers (1). Owing to earlier and more accurate diagnosis, as well as increased life expectancy or improved survival, the global prevalence of MS has been well-documented to have increased in the last few decades (2, 3). Global estimates indicate that there were approximately 2.2 million prevalent MS cases in 2016, which rose to 2.8 million in 2020 (2, 3). Numerous studies have also highlighted significant disparities in MS prevalence based on geographical region, race, ethnicity, age, and sex (2, 3). The estimated prevalence of MS ranges from 2 per 100,000 person in Asia to approximately 100 per 100,000 individuals in Western countries (4). Usually, white individuals of European descent and females typically have a higher risk of developing MS. However, the incidence of MS has recently been on the rise among the black population in the USA, particularly in black women, which suggests that both environmental and genetic factors have undergone changes (5).

MS mainly affects young adults aged 20–40 years and is characterized by chronic progression and increasing disability over time (6, 7). Disability progression may not be clinically apparent until decades later, despite neurodegenerative processes start at disease onset. Advancements in disease-modifying therapies (DMTs) greatly alleviate the progression of disease to disability among patients with multiple sclerosis (8). However, the average age of MS onset is increasing, with approximately 9–25% of all MS patients being older than 65 years (9, 10). Age is strongly associated with the clinical course of MS, and those with later-onset disease face a more rapid development of permanent disability (11). Aging is also associated with increased risks of side-effects caused by some MS therapies. Increasing large-scale cohort studies have reported a decline in the efficacy of disease-modifying therapies with advancing age (12). Nevertheless, the safety and efficacy of disease-modifying therapies in the elderly population are understudied, and consequently, very little is known (13). Therefore, managing elder population with MS presents unique challenges related to age-related and disease-related comorbidities, as well as the disease itself (9).

Population aging is most pronounced in developed countries. At the same time, regions with a high Socio-demographic Index (SDI) often experience a greater burden of Multiple Sclerosis (MS) (14). Additionally, this age group often transitions from being net contributors to the economy to net recipients of social security and pension benefits. Despite two convincing longitudinal population-based studies in Northern Europe reporting increased survival rates among MS patients over the past 60 years (15, 16), MS patients still have a life expectancy that is 7 years shorter and a mortality rate almost three times higher than the general population (16). Additionally, a nested case–control study from a large cohort study conducted in the USA revealed higher mortality rate and comorbidities in MS patients compared to matched controls without MS (17). As the average age of the MS population increases, the physiological effects of aging, along with the pathological and immunological changes linked to aging and disease progression, it is essential to fully comprehend the burden of MS among older adults, which is essential for planning and allocating healthcare resources efficiently and inform interventions to enhance their well-being (18).

Smoking is recognized as a major environmental risk factor for MS, influencing both the disease’s onset and progression (19). This association is thought to arise from smoking-induced inflammation and immune system modulation (20). MS patients who smoke may have a reduced therapeutic response, resulting in worse clinical outcomes (21). Smoking prevalence among older adults is generally lower than in younger age groups, but it remains a significant public health concern (22). For example, in 2018, about 11.2% of adults aged 65 and older in the United States were current smokers (23), and in China, there were more than 38 million older smokers (24). While MS mainly affects women, men generally have higher smoking rates than women. Furthermore, in a Swedish cohort of newly diagnosed MS cases, nearly 60% of the patients were smokers, highlighting smoking as a significant factor influencing patient outcomes (25). Therefore, it is important to investigate the proportion of the MS burden attributable to smoking worldwide. The Global Burden of Disease (GBD) study systematically measures the incidence, prevalence, mortality, and resulting health loss caused by diseases and injuries (26). This study utilized the GBD study 2019 data to provide global, regional, and national-level estimates of MS burden among older adults aged 65–89 years and its temporal trends between 1990 and 2019, while considering variations in region, time, gender, and the socio-demographic index (SDI).

## 2 Methods

### 2.1 Data source and data collection

The GBD study, a multinational collaborative research study led by the Institute for Health Metrics and Evaluation (IHME), aims to identify, compile and standardize health loss caused by diseases, injuries, and risk factors across different age groups, sexes, and geographic regions at specific points in time. The GBD study 2019 provides insights into the age-specific and sex-specific estimates of incidence, prevalence, mortality, Years of life lost (YLL), Years lived with disability (YLD), and Disability-adjusted Life Years (DALYs) for 369 diseases and injuries and 87 risk factors in 204 countries and territories (26). DALYs are a measure that combines the years of potential life lost due to premature death (YLL) and the years of productive life lost due to disability (YLD) when compared to a standardized life expectancy. This measure enables a comprehensive assessment of disease burden, capturing the cumulative number of years lost to ill-health, disability, or premature death. Detailed information about the GBD study protocol can be accessed online.^1^ In this cross-sectional study, we collected annual estimates of SDI-, region-, country-, and age-specific numbers and crude rates of YLL and YLD for MS from the GBD Study 2019 using the Global Health Data Exchange (GHDx) query tool.^2^ The study included individuals aged 65–89 years with MS across 204 countries from 1990 to 2019, dividing the population into five-year age brackets: 65–69, 70–74, 75–79, 80–84, and 85–89 years. This dataset included information on YLL and YLD of MS among older adults aged 65–89 years as well as smoking-attributable MS burden in this population.

In the GBD study, the diagnosis of MS is based on the McDonald criteria (27) and is identified by the International Classification of Diseases 10th revision (ICD-10) code G35.0. For the GBD study 2019, the data underpinning estimates of burden due to MS are generally of two types. The first are representative, population-based, cross-sectional or longitudinal studies reported in peer-reviewed journals and identified via a search-string-based review. The second type are claims data as obtained and processed by the GBD Clinical Informatics team. A pre-modelling bias adjustment was then made to data from USA claims in the year 2000—a dataset that only covers a small commercially insured sub-population. This adjustment was modelled as difference in logit prevalence between USA claims data and reference data matched on year, age, sex and location. The estimated mean logit differences were applied to the USA claims data for 2000 prior to modelling in DisMod-MR 2.1 (26). Moreover, smoking (defined as any previous or current tobacco smoking) attributable MS burden was estimated in the GBD study (26).

Furthermore, GBD study researchers have also developed the SDI as a composite indicator of development status. The SDI consists of three key metrics: the total fertility rate under the age of 25, the mean education for those ages 15 and older, and the lag distributed income *per capita*. These metrics offer insights into the social and economic conditions within a region or country, which are strongly correlated with health outcomes. Based on the SDI quintiles, which is calculated as the geometric mean ranging from 0 to 1, regions and countries are categorized into five quintiles: low, low-middle, middle, high-middle, and high. This categorization enables a better understanding of the development status and potential disparities across different areas.

### 2.2 Years of life lost and years lived with disability

YLL is a valuable measure for public health surveillance, particularly for quantifying the level and trends of premature mortality, identification of leading causes of premature deaths and monitoring the progress of YLL as a key indicator of population health (28). YLL due to premature mortality, as developed by the GBD study, overcomes the issue related to the arbitrary selection of age threshold by basing its metrics on time lost rather than number of deaths, and calculating time lost based on the potential maximum life span of an individual at each age. YLL is being systematically estimated by the GBD study to support public health planning and guide public health policy and programs at global, national and subnational levels (26). Years lived with disability (YLD) reflect the burden of non-fatal health loss, which is estimated through the number of prevalence cases multiplied by the corresponding disability weights (the severity of health loss associated with a health state that is developed through surveys of the general public) for that condition (29). The sum of YLL and YLD equals the disability-adjusted life years (DALYs).

### 2.3 Statistical analysis

In this study, the age-standardized rate (ASR) and the estimated annual percentage change (EAPC) in ASR were employed to measure the distribution and trends in disease burden of MS among older adults (30). These measures provide a standardized assessment of the disease burden across different locations. Standardization is necessary when comparing multiple populations with varying age structures or when examining changes within a single population over time, as age profiles fluctuate accordingly. The ASR (per 100,000) was calculated using the following formula, where $a_{i}$ denotes the age-specific rate in the $i^{th}$ age subgroup and $w_{i}$ represents the number of individuals in the same age class of the selected reference standard population.


$$\mathrm{ASR}=\frac{\sum_{i=1}^{A}a_{i}w_{i}}{\sum_{i=1}^{A}w_{i}}\times 100,000$$

Moreover, EAPC was widely applied to quantify and summarize the temporal trend of ASR (31). A regression line was fitted to the natural logarithm of the rates, denoted as y=α+βx+ε , where y=ln(ASR) , and x=calendaryear . The EAPC was calculated as 100×(exp(β)−1) , and the corresponding 95% confidence interval (CI) could be obtained from the linear regression model. If both the EAPC value and the lower limits of its 95% CI were greater than 0, the ASR was considered to exhibit an increasing trend; conversely, if both were less than 0, a decreasing trend was observed. Moreover, Pearson correlation analysis was conducted to assess the association between ASRs and SDI level, as well as EAPC of ASRs and SDI level.

Furthermore, the age-standardized YLD and YLL rates of MS among older adults from 2020 to 2040 were projected by used the Bayesian age-period-cohort (BAPC) model integrating nested Laplace approximations (32). Standard Populations data came from the World Standards database,^3^ and population forecast data was collected from the GBD Study 2019 Global Fertility, Mortality, Migration, and Population Forecasts 2017–2,100 (33). The BAPC model is a method for analyzing and predicting trends in disease burden by applying Bayesian formulas to calculate hypothetical probability distributions based on 3 factors: age, period, and cohort and combining *a priori* and sample information to derive posterior information. In the BAPC model, the prior probability distribution of time period and birth cohort effects served as prior information, while the effects of age, period, and cohort were estimated through a random walk of varying orders (32). Compared with methods that estimate the overall parameters from sample statistics only, BAPC is more flexible in the choice of parameters and prior probability distributions, and the predictions are more robust and reliable (34).

All statistics Data analysis was conducted using R software, version 4.1.0 (R Foundation for Statistical Computing). A two-tail *p* value <0.05 was considered statistically significant.

## 3 Results

### 3.1 Global overall and smoking-attributable burden of MS among older adults

**Figure 1:**
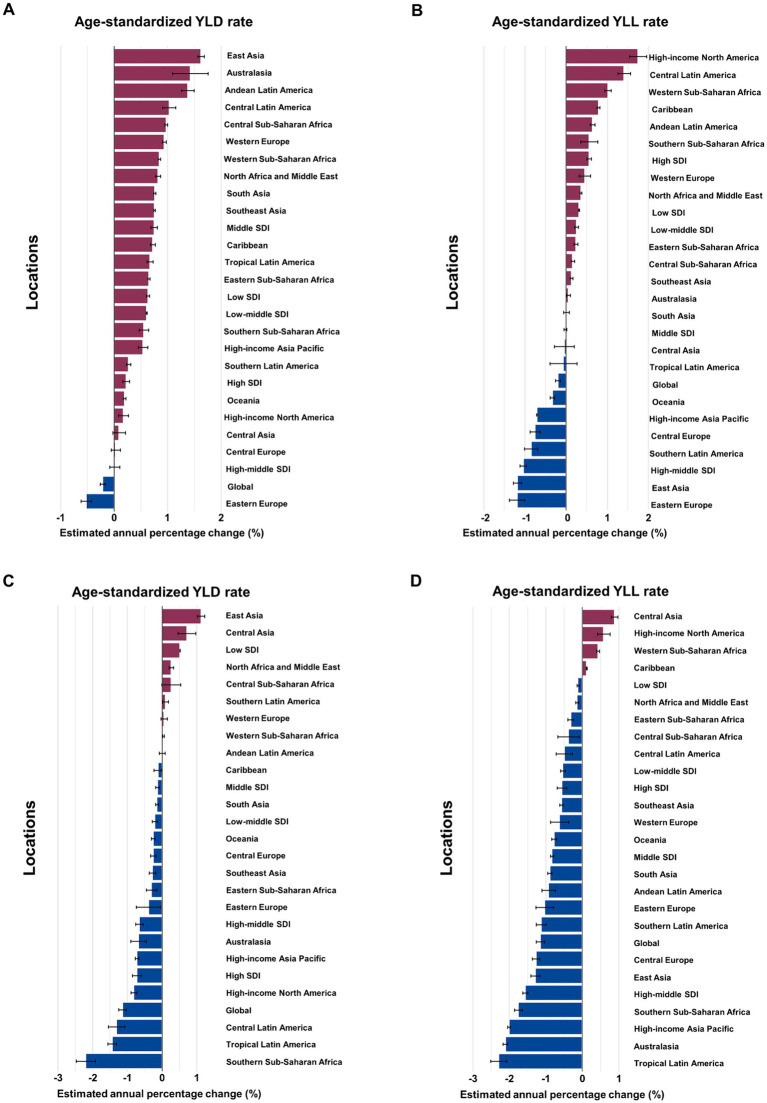
Estimated annual percentage change (EAPC) of age-standardized YLD and YLL rates for overall and smoking-attributable multiple sclerosis among older adults aged 65–89 years by location, 1990–2019. **(A)** EAPC of age-standardized YLD rate for MS. **(B)** EAPC of age-standardized YLL rate for MS. **(C)** EAPC of age-standardized YLD rate for MS attributable to smoking. **(D)** EAPC of age-standardized YLL rate for MS attributable to smoking. YLD, years of life lived with disability; YLL, years of life lost.

In 2019, the number of YLD due to MS among older adults aged 65–89 years worldwide was 80,040 (95% UI: 57,534 to 103,608), with an age-standardized rate of 11.43 YLD (95% UI: 11.36 to 11.51) per 100,000 population (Table 1). The age-standardized YLD rate of MS decreased by an average of −0.21% (95% CI –0.26 to −0.16) per year globally between 1990 and 2019 (Figure 1A). Globally, an estimated 139,132 (95% UI: 107,632 to 161,172) YLL caused by MS were reported in 2019, with an age-standardized rate of 19.95 YLL (95% UI: 19.85 to 20.05) per 100,000 population. The age-standardized YLL rate of MS tends to trend downward, with an average change of −0.2% (95% CI: −0.26 to −0.14) per year globally (Figure 1B).

**Table 1:** The YLD and YLL of multiple sclerosis in older adults aged 65–89 years in 2019.

Characteristics	YLD		YLL	
	Case No. (95% UI)	ASR per 100,000 No. (95% UI)	Case No. (95% UI)	ASR per 100,000 No. (95% UI)
Overall	80,040 (57,534 to 103,608)	11.43 (11.36 to 11.51)	139,132 (107,632 to 161,172)	19.95 (19.85 to 20.05)
Sex				
Male	24,135 (17,296 to 31,524)	7.52 (7.43 to 7.6)	54,417 (40,530 to 67,595)	16.88 (16.75 to 17.02)
Female	55,905 (40,306 to 72,324)	14.76 (14.64 to 14.87)	84,715 (56,292 to 104,262)	22.62 (22.47 to 22.76)
Socio-demographic index				
High	49,100 (35,580 to 63,160)	29.11 (28.86 to 29.35)	82,469 (49,528 to 93,331)	50.11 (49.78 to 50.45)
High-middle	18,185 (13,052 to 23,712)	10.12 (9.98 to 10.25)	24,645 (20,172 to 38,592)	13.8 (13.64 to 13.96)
Middle	7,525 (5,200 to 10,051)	3.7 (3.63 to 3.77)	16,762 (14,438 to 21,204)	8.24 (8.13 to 8.36)
Low-middle	3,947 (2,675 to 5,414)	3.61 (3.52 to 3.7)	11,368 (9,357 to 13,529)	10.37 (10.2 to 10.54)
Low	1,241 (824 to 1703)	3.27 (3.13 to 3.4)	3,821 (2,685 to 5,037)	10.02 (9.76 to 10.28)
Region				
High-income Asia Pacific	2,132 (1,435 to 2,929)	5.08 (4.9 to 5.25)	2,181 (1720 to 3,506)	5.26 (5.09 to 5.44)
Central Asia	1,075 (762 to 1,432)	21.44 (20.5 to 22.39)	675 (563 to 925)	14.47 (13.69 to 15.24)
East Asia	1,582 (1,017 to 2,237)	0.86 (0.83 to 0.9)	9,307 (7,558 to 12,350)	5.07 (4.98 to 5.16)
South Asia	3,855 (2,534 to 5,393)	3.4 (3.31 to 3.48)	12,284 (9,975 to 15,027)	10.81 (10.64 to 10.99)
Southeast Asia	413 (261 to 592)	0.9 (0.84 to 0.96)	3,019 (2,365 to 4,270)	6.62 (6.43 to 6.81)
Australasia	1,138 (771 to 1,564)	25.56 (24.4 to 26.73)	1819 (1,224 to 2,349)	41.22 (39.74 to 42.7)
Caribbean	215 (143 to 297)	4.88 (4.46 to 5.29)	551 (392 to 673)	12.85 (12.1 to 13.61)
Central Europe	2,655 (1855 to 3,433)	13.07 (12.67 to 13.47)	7,219 (5,586 to 11,561)	35.92 (35.18 to 36.66)
Eastern Europe	1992 (1,351 to 2,776)	6.43 (6.21 to 6.65)	4,229 (2,773 to 8,842)	13.7 (13.32 to 14.07)
Western Europe	30,684 (21,697 to 40,103)	38.07 (37.67 to 38.47)	43,832 (28,796 to 52,117)	57 (56.48 to 57.51)
Andean Latin America	186 (126 to 257)	4 (3.64 to 4.37)	415 (305 to 519)	9.04 (8.43 to 9.64)
Central Latin America	779 (524 to 1,069)	4.07 (3.86 to 4.28)	2,482 (1904 to 3,064)	13.02 (12.6 to 13.43)
Southern Latin America	786 (533 to 1,102)	10.69 (10.15 to 11.22)	1,110 (877 to 1866)	15.24 (14.55 to 15.94)
Tropical Latin America	2,336 (1,598 to 3,131)	11.67 (11.28 to 12.05)	2,512 (2,109 to 3,595)	12.45 (12.04 to 12.86)
North Africa and Middle East	6,335 (4,557 to 8,249)	20.21 (19.79 to 20.63)	4,325 (3,664 to 5,458)	13.72 (13.38 to 14.06)
High-income North America	23,063 (16,851 to 29,617)	40.95 (40.46 to 41.44)	39,855 (21,729 to 46,439)	71.54 (70.87 to 72.21)
Oceania	2 (1 to 3)	0.47 (0.47 to 0.47)	22 (15 to 32)	4.82 (4.82 to 4.83)
Central Sub-Saharan Africa	54 (34 to 77)	1.44 (1.21 to 1.67)	236 (143 to 338)	6.61 (6.06 to 7.16)
Eastern Sub-Saharan Africa	207 (133 to 294)	1.77 (1.61 to 1.92)	838 (450 to 1,196)	7.3 (6.94 to 7.66)
Southern Sub-Saharan Africa	158 (104 to 221)	3.61 (3.26 to 3.97)	390 (321 to 452)	9.15 (8.5 to 9.79)
Western Sub-Saharan Africa	393 (266 to 544)	2.98 (2.78 to 3.19)	1832 (1,474 to 2,552)	14.13 (13.62 to 14.64)

YLL, years of life lost; YLD, years of life lived with disability; ASR, age-standardized rate.

Globally, the number of YLD due to MS attributable to smoking reported among older adults aged 65–89 years in 2019 was 7,891 (95% UI: 5,003 to 10,991), with an age-standardized rate of 1.13 YLD (95% UI: 1.11 to 1.15) per 100,000 population (Table 2). The age-standardized rate of YLD decreased by an average of −1.14% (95% CI: −1.25 to −1.04) annually from 1990 to 2019 (Figure 1C). The number of YLL for smoking-attributable MS globally was 15,667 (95% UI: 10,833 to 20,076) in 2019, with an age-standardized rate of 2.25 DALYs (95% UI: 2.22 to 2.29) per 100,000. The age-standardized rate of YLL due to smoking-attributable MS decreased by an annual average of −1.15% (95% CI: −1.27 to −1.03) globally from 1990 to 2019 (Figure 1D).

**Table 2:** The YLD and YLL of multiple sclerosis attributable to smoking in older adults aged 65–89 years in 2019.

Characteristics	YLD		YLL	
	Case No. (95% UI)	ASR per 100,000 No. (95% UI)	Case No. (95% UI)	ASR per 100,000 No. (95% UI)
Overall	7,891 (5,003 to 10,991)	1.13 (1.11 to 1.15)	15,667 (10,833 to 20,076)	2.25 (2.22 to 2.29)
Sex				
Male	3,735 (2,380 to 5,268)	1.15 (1.12 to 1.18)	8,947 (6,320 to 12,409)	2.76 (2.71 to 2.81)
Female	4,156 (2,636 to 5,767)	1.12 (1.09 to 1.15)	6,720 (3,691 to 9,099)	1.82 (1.78 to 1.86)
Socio-demographic index				
High	5,344 (3,367 to 7,473)	3.29 (3.22 to 3.37)	9,744 (5,585 to 12,990)	6.09 (5.99 to 6.2)
High-middle	1,634 (1,058 to 2,324)	0.92 (0.89 to 0.95)	2,823 (1961 to 4,720)	1.59 (1.54 to 1.64)
Middle	580 (360 to 834)	0.28 (0.26 to 0.3)	1852 (1,278 to 2,750)	0.9 (0.87 to 0.93)
Low-middle	261 (155 to 394)	0.23 (0.22 to 0.25)	1,007 (684 to 1,364)	0.9 (0.86 to 0.95)
Low	68 (41 to 104)	0.17 (0.15 to 0.19)	233 (146 to 328)	0.59 (0.54 to 0.64)
Region				
High-income Asia Pacific	163 (99 to 249)	0.42 (0.37 to 0.46)	235 (161 to 405)	0.6 (0.55 to 0.65)
Central Asia	96 (59 to 141)	1.83 (1.6 to 2.06)	53 (34 to 85)	1.05 (0.89 to 1.21)
East Asia	217 (130 to 333)	0.12 (0.11 to 0.13)	1,678 (1,146 to 2,572)	0.9 (0.87 to 0.94)
South Asia	242 (141 to 373)	0.21 (0.19 to 0.23)	961 (615 to 1,352)	0.83 (0.79 to 0.87)
Southeast Asia	35 (21 to 53)	0.07 (0.05 to 0.08)	365 (249 to 552)	0.78 (0.73 to 0.84)
Australasia	69 (41 to 103)	1.56 (1.35 to 1.78)	117 (75 to 165)	2.63 (2.33 to 2.94)
Caribbean	16 (9 to 24)	0.38 (0.37 to 0.38)	51 (30 to 72)	1.14 (0.96 to 1.33)
Central Europe	311 (195 to 435)	1.55 (1.43 to 1.67)	897 (604 to 1,541)	4.53 (4.3 to 4.75)
Eastern Europe	184 (112 to 273)	0.59 (0.53 to 0.65)	416 (236 to 969)	1.35 (1.25 to 1.45)
Western Europe	3,121 (1960 to 4,408)	4.16 (4.04 to 4.28)	5,101 (3,206 to 6,823)	6.98 (6.81 to 7.14)
Andean Latin America	5 (3 to 7)	0.1 (0.1 to 0.1)	17 (10 to 25)	0.37 (0.37 to 0.37)
Central Latin America	31 (18 to 49)	0.17 (0.17 to 0.17)	137 (86 to 194)	0.69 (0.62 to 0.77)
Southern Latin America	69 (39 to 107)	0.96 (0.83 to 1.1)	107 (67 to 201)	1.48 (1.31 to 1.66)
Tropical Latin America	201 (119 to 300)	0.99 (0.9 to 1.08)	221 (148 to 336)	1.07 (0.97 to 1.17)
North Africa and Middle East	472 (295 to 683)	1.47 (1.38 to 1.56)	400 (268 to 592)	1.24 (1.16 to 1.32)
High-income North America	2,635 (1,660 to 3,755)	4.73 (4.58 to 4.87)	4,791 (2,491 to 6,574)	8.68 (8.47 to 8.89)
Oceania	0	0.04 (0.03 to 0.04)	2 (1 to 3)	0.43 (0.43 to 0.43)
Central Sub-Saharan Africa	1 (1 to 2)	0.04 (0.04 to 0.04)	8 (4 to 14)	0.22 (0.22 to 0.22)
Eastern Sub-Saharan Africa	8 (4 to 12)	0.07 (0.07 to 0.07)	39 (18 to 66)	0.3 (0.24 to 0.36)
Southern Sub-Saharan Africa	8 (5 to 13)	0.18 (0.18 to 0.18)	26 (17 to 35)	0.53 (0.41 to 0.65)
Western Sub-Saharan Africa	8 (5 to 12)	0.06 (0.06 to 0.06)	44 (28 to 65)	0.31 (0.26 to 0.37)

YLL, years of life lost; YLD, years of life lived with disability; ASR, age-standardized rate.

### 3.2 Regional level overall and smoking-attributable burden of MS among older adults

In term of GBD regions, the High-income North America and Western Europe had the largest burden of overall and smoking-attributable MS among older adults in 2019 (Tables 1, 2). Between 1990 and 2019, the largest increases in age-standardized YLD rate of MS occurred in East Asia (average annual change 1.62% [95% CI: 1.56 to 1.68]), Australasia (1.42% [1.09 to 1.76]), and Andean Latin America (1.38% [1.09 to 1.76]; Figure 1A). In the same period, the largest increases in age-standardized YLL rate were found in High-income North America (1.74% [1.53 to 1.96]), and Central Latin America (1.41% [1.26 to 1.56]; Figure 1B). From 1990 to 2019, East Asia (1.12% [1.01 to 1.23]) and Central Asia (0.88% [0.79 to 0.97]) had the greatest increase in age-standardized rates of YLD and YLL for smoking-attributable MS among the elderly adults, respectively (Figures 1C,D).

### 3.3 National level overall and smoking-attributable burden of MS among older adults

**Figure 2:**
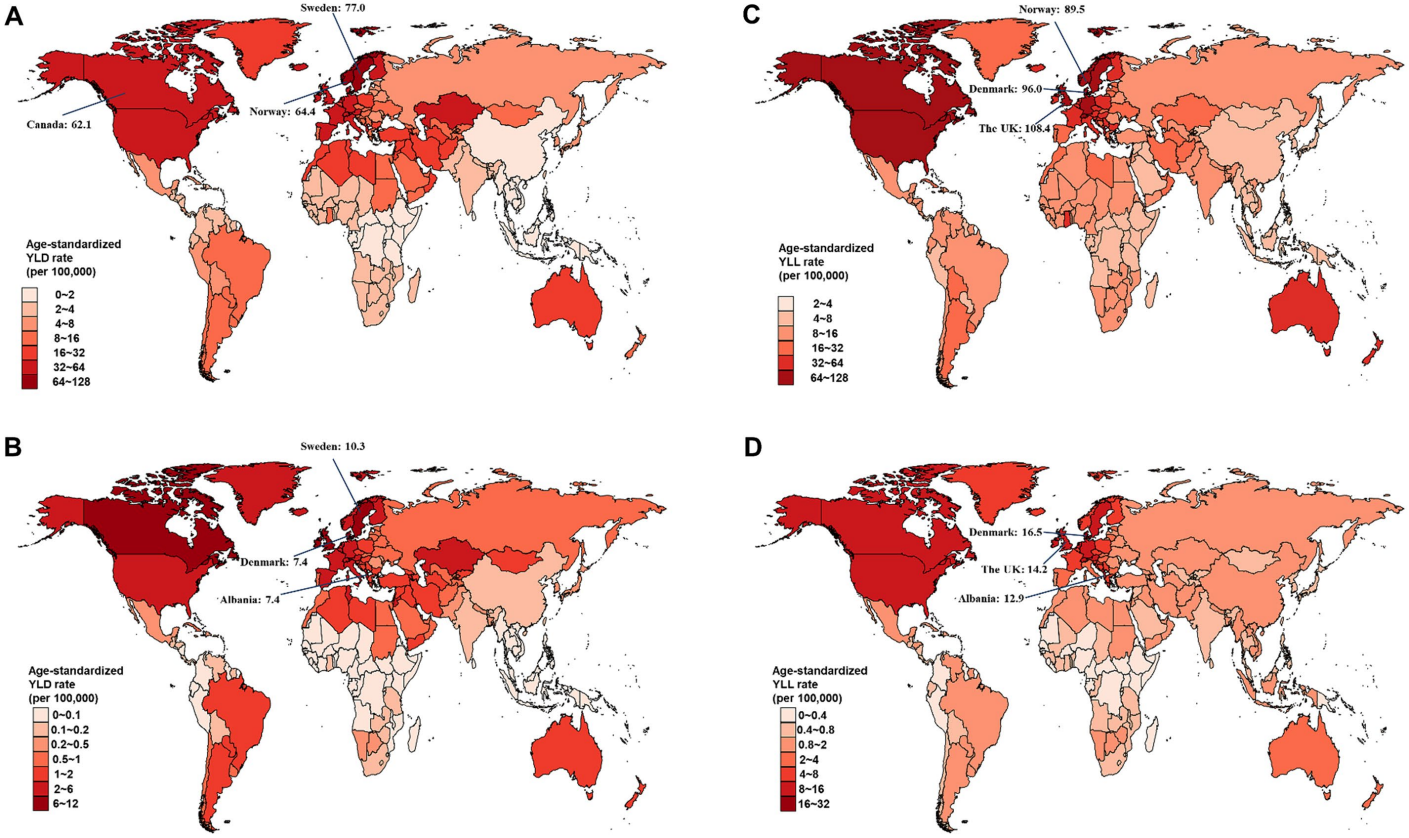
Estimated age-standardized rates of YLD and YLL for overall and smoking-attributable multiple sclerosis among older adults aged 65–89 years from 1990 to 2019, by country. **(A)** Age-standardized YLD rate of MS; **(B)** Age-standardized YLD rate of smoking-attributable MS; **(C)** Age-standardized YLL rate of MS; **(D)** Age-standardized YLL rate of smoking-attributable MS. YLD, years of life lived with disability; YLL, years of life lost.

**Figure 3:**
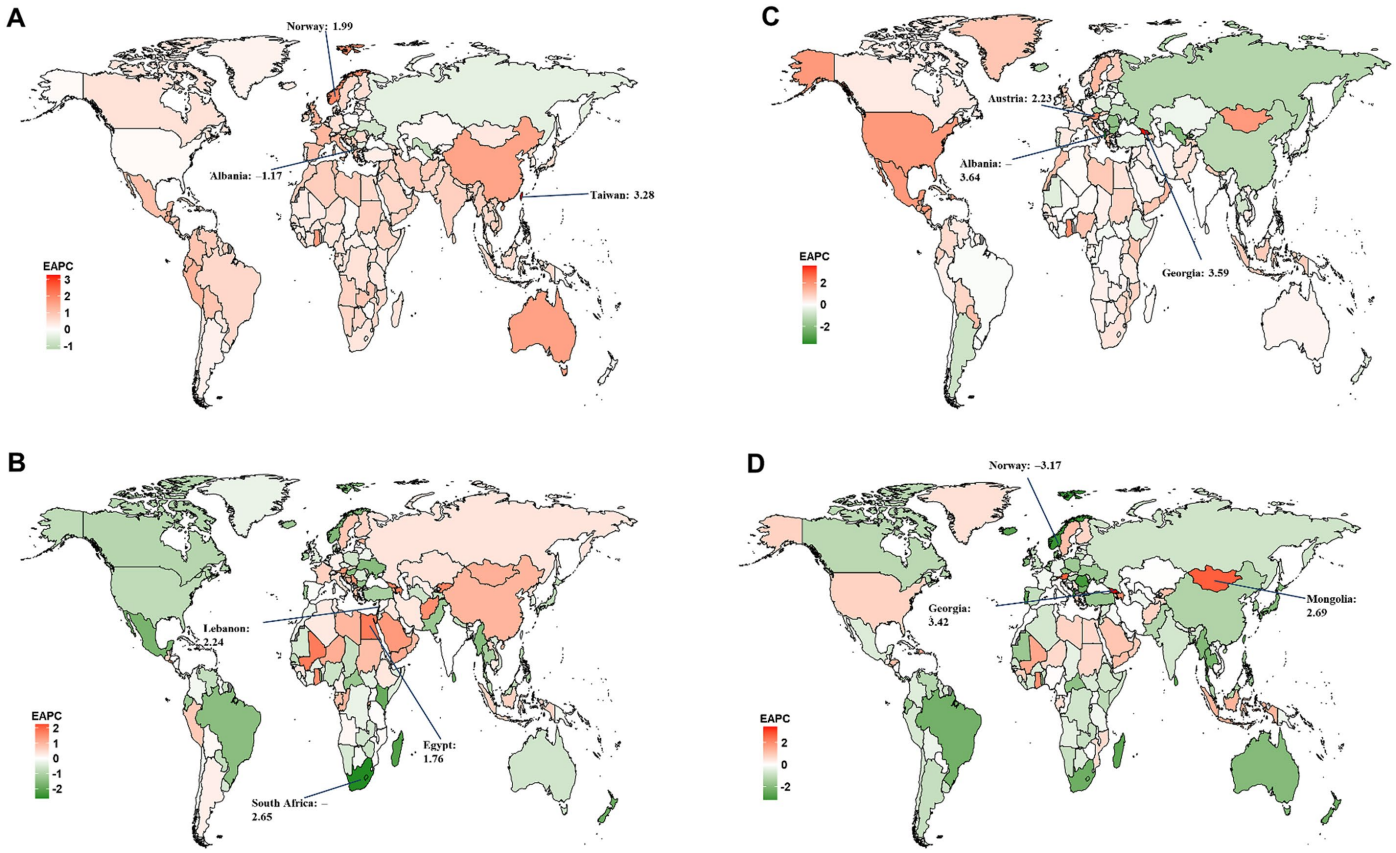
Estimated annual percentage change (EAPC) of the age-standardized rates of YLD and YLL for overall and smoking-attributable multiple sclerosis among older adults aged 65–89 years from 1990 to 2019, by country. **(A)** EAPC of age-standardized YLD rate for MS; **(B)** EAPC of age-standardized YLD rate for MS attributable to smoking; **(C)** EAPC of age-standardized YLL rate for MS; **(D)** EAPC of age-standardized YLL rate for MS attributable to smoking. YLD, years of life lived with disability; YLL, years of life lost.

At the national level, Sweden had the largest age-standardized YLD rate for overall and smoking-attributable MS among older adults aged 65–89 years in 2019 (Figures 2A,B). The highest age-standardized YLL rate for both overall and smoking-attributable MS were found in United Kingdom and Denmark (Figures 2C,D). From 1990 to 2019, Taiwan (Province of China) and Lebanon had the fastest increase in age-standardized YLD rate of overall and smoking-attributable MS, respectively (Figures 3A,B). Additionally, the largest increase in age-standardized YLL rate for both overall and smoking-attributable MS was observed in Georgia (Figures 3C,D).

**Figure 4:**
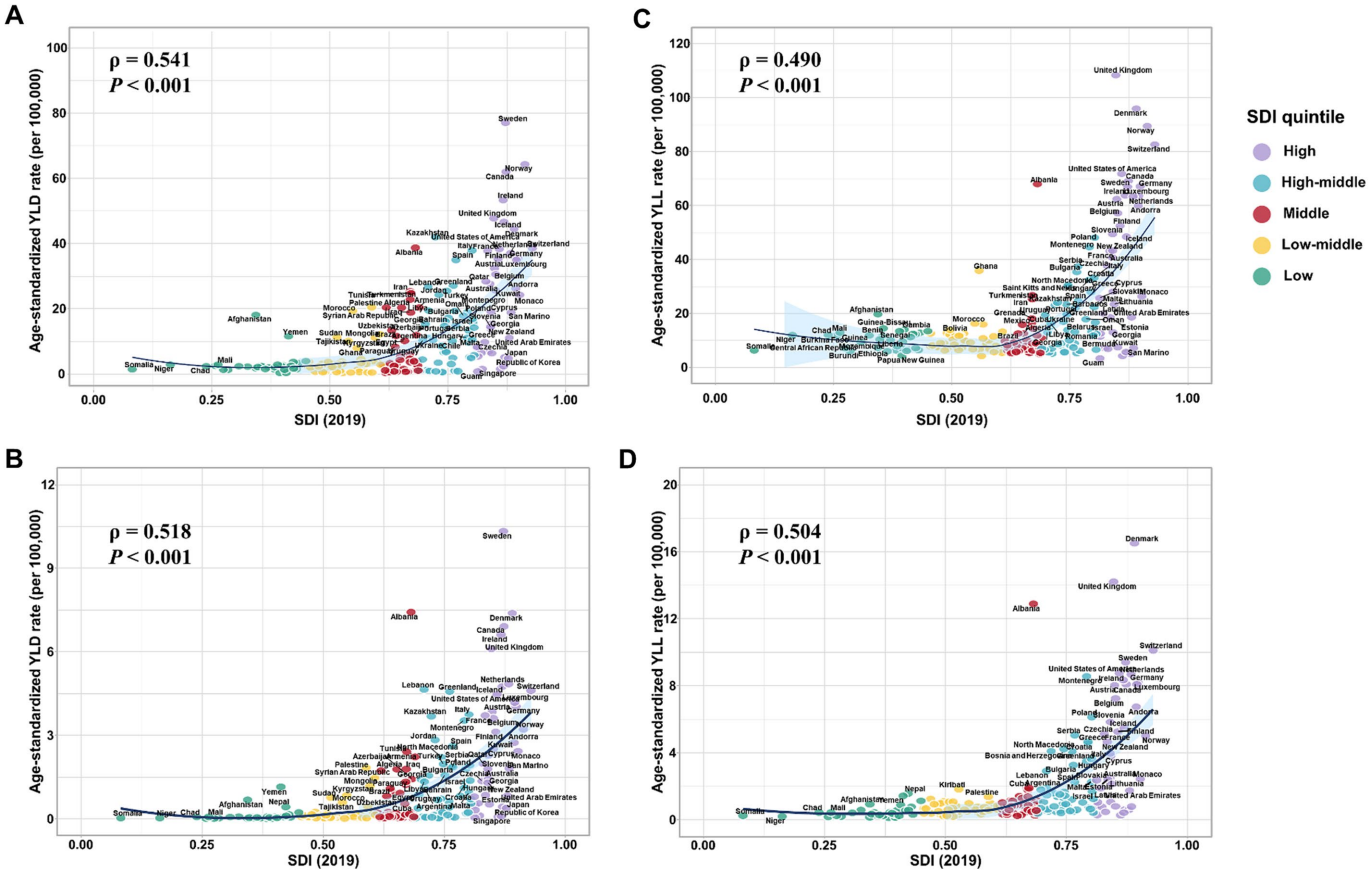
Estimated age-standardized rates of YLD and YLL for overall and smoking-attributable multiple sclerosis among older adults aged 65–89 years in 2019 versus the 2019 SDI level. **(A)** Age-standardized YLD rate of MS versus the 2019 SDI level; **(B)** Age-standardized YLD rate of smoking-attributable MS versus the 2019 SDI level; **(C)** Age-standardized YLL rate of MS versus the 2019 SDI level; **(D)** Age-standardized YLL rate of smoking-attributable MS versus the 2019 SDI level. YLD, years of life lived with disability; YLL, years of life lost; SDI, socio-demographic index.

Our results show the national level age-standardized YLD and YLL rates of both overall and smoking-attributable MS among older adults aged 65–89 years increased exponentially with increases in SDI level (all model *p* < 0.001; Figure 4). However, the correlations between the 2019 SDI level and the EAPC of the age-standardized rates of YLD and YLL for overall and smoking-attributable multiple sclerosis from 1990 to 2019 were not significant (Supplementary Figure S1).

### 3.4 Overall and smoking-attributable burden of MS among older adults by SDI quintile

In 2019, the highest number and age-standardized rates of YLD and YLL for overall and smoking-attributable MS among older adults were seen in the high SDI quintile (Tables 1, 2). From 1990 to 2019, the age-standardized YLD rate for MS increased across all 5 SDI quintiles with the middle SDI region experienced the fastest increase (EAPC =0.75, 95% CI: 0.69 to 0.8; Figure 1A). However, the high SDI quintile experienced the largest increase in the age-standardized YLL rate for MS among older adults (EAPC =0.55, 95% CI: 0.5 to 0.61; Figure 1B). For smoking-attributable MS burden in the elderly population by SDI quintile, the fastest reductions of age-standardized rates of YLD and YLL were observed in the high SDI quintile (EAPC = −0.73, 95% CI: −0.86 to −0.6) and the high-middle SDI quintile (EAPC = −1.56, 95% CI: −1.63 to −1.49), respectively, between 1990 and 2019 (Figures 1C,D). Conversely, regions with low SDI level showed an increased trend in the age-standardized YLD rate and the lowest decrease in age-standardized YLL rate for MS attributable to smoking. Moreover, the percent contribution of YLDs in DALYs of both overall and smoking-attributable MS among older adults increased across SDI quintiles except for the high SDI quintile (Supplementary Figure S2).

### 3.5 Sex difference of overall and smoking-attributable burden of MS among older adults

**Figure 5:**
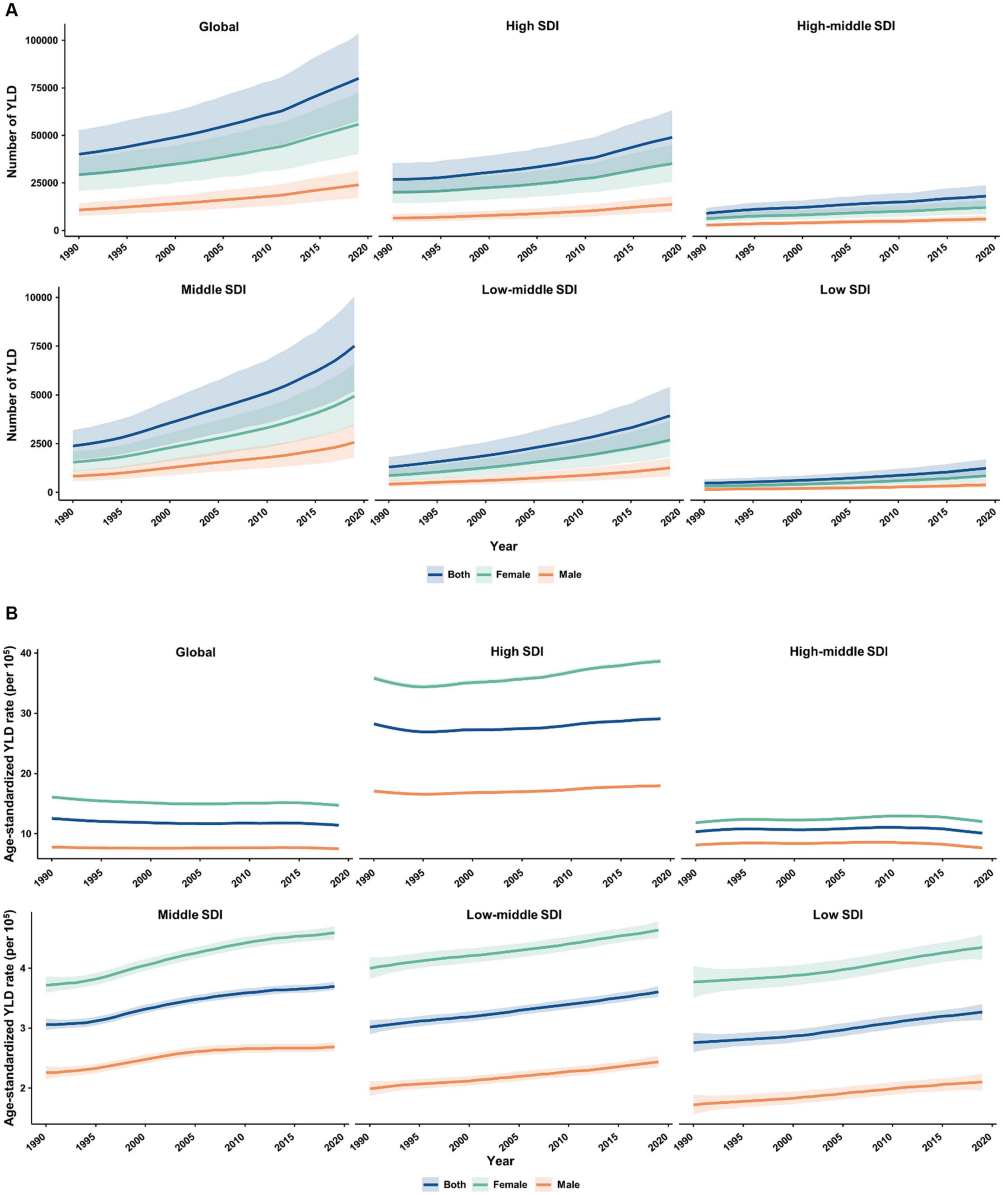
Temporal patterns of YLD caused by multiple sclerosis in older adults aged 65–89 years worldwide and in 5 SDI quintiles, 1990–2019. **(A)** The number of YLD; **(B)** Age-standardized YLD rate (per 100,000). YLD, years of life lived with disability; SDI, socio-demographic index.

**Figure 6:**
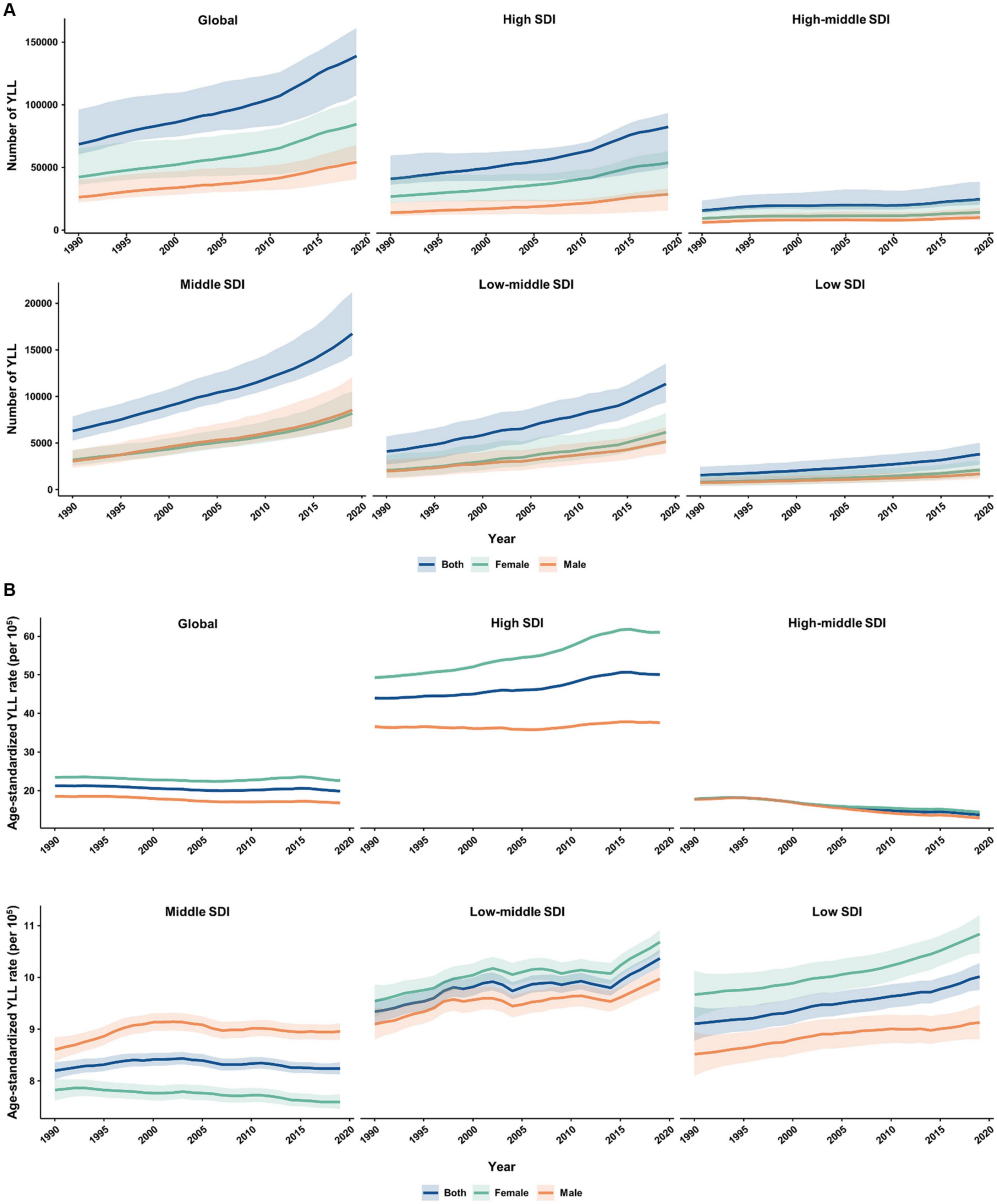
Temporal patterns of YLL caused by multiple sclerosis in older adults aged 65–89 years worldwide and in 5 SDI quintiles, 1990–2019. **(A)** The number of YLL; **(B)** Age-standardized YLL rate (per 100,000). YLL, years of life lost; SDI, socio-demographic index.

From 1990 to 2019, the absolute number and age-standardized rate of YLD for MS in the elderly population were generally higher among females than males worldwide and across 5 SDI quintiles (Figure 5). The number of YLL due to MS was similar between females and males in all SDI quintiles, except for the high SDI quintile where it was higher in females (Figure 6A). Additionally, females had significantly higher age-standardized YLL rate of MS than males in the high, low-middle, and low SDI regions. However, the age-standardized YLL rate of MS was higher in males than females in the middle SDI quintile (Figure 6B).

Globally, the absolute number and age-standardized rate of YLD for smoking-attributable MS in the elderly population were similar between females and males (Supplementary Figure S3). However, males had significantly larger number and age-standardized rate of YLD for smoking-attributable MS than females across SDI regions (barring the high SDI region). Conversely, across the globe and SDI regions (except for the high SDI region), males had higher number and age-standardized rate of YLD for MS attributable to smoking (Supplementary Figure S4).

### 3.6 Age pattern of overall and smoking-attributable burden of MS among older adults

Globally, the age-specific rate of YLD for MS is similar across the five older adult age groups from 1990 to 2019 (Supplementary Figures S5, S6). However, in high-SDI regions such as Australasia, Western Europe, and High-income North America, the age-specific rate of YLD significantly decreases with increasing age (Supplementary Figure S6). Between 1990 and 2019, the age-specific rate of YLL for MS is highest among individuals aged 65–69 years, a trend also observed for the age-specific rates of YLD and YLL for MS due to smoking (Supplementary Figures S7–S9).

Overall, the proportion of the MS burden attributable to smoking declined across the five older adult age groups from 1990 to 2019 (Supplementary Figures S10, S11). The highest fractions of MS burden attributable to smoking were observed in individuals aged 65–69 years, decreasing with age, except for MS YLL in Southeast Asia, which showed a reverse trend (Supplementary Figure S11). Globally, smoking was responsible for 15.84 and 10.36% of MS YLD in the 65–69 age group in 1990 and 2019, respectively, and for 17.57 and 11.58% of MS YLL in 1990 and 2019. Furthermore, the impact of smoking varied by region, with higher-SDI regions having a higher proportion of the MS burden attributable to smoking (Supplementary Figures S10, S11).

### 3.7 Prediction of the age-standardized rates of YLD and YLL for overall and smoking-attributable MS among older adults worldwide

**Figure 7:**
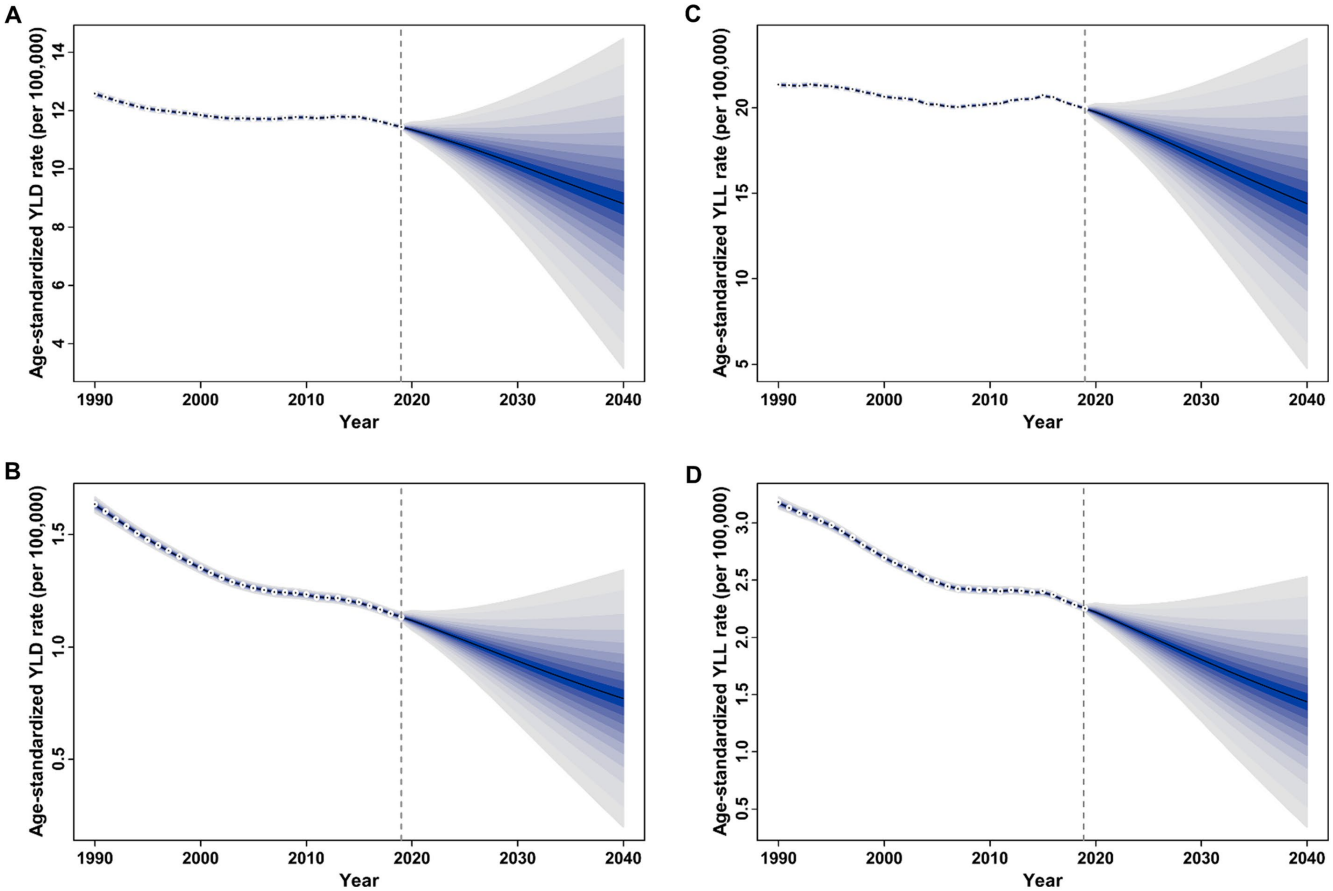
Trends of age-standardized rate of YLD and YLL for overall and smoking-attributable multiple sclerosis among older adults aged 65–89 years worldwide: observed rate (1990–2019) and predicted rates (2020–2040). **(A)** Age-standardized YLD rate of MS; **(B)** Age-standardized YLD rate of smoking-attributable MS; **(C)** Age-standardized YLL rate of MS; **(D)** Age-standardized YLL rate of smoking-attributable MS. The blue region in shows the upper and lower limits of the 95% uncertainty interval (UI). YLD, years of life lived with disability; YLL, years of life lost.

Figure 7 displays the projected trends for age-standardized YLD and YLL rates of overall and smoking-attributable MS among older adults aged 65–89 years globally from 2020 to 2040. Generally, the global age-standardized YLD rate of both overall and smoking attributable MS is expected to decrease from 11.34 and 1.12 per 100,000 populations in 2020 to 8.81 and 0.77 per 100,000 populations in 2040, respectively (Figures 7A,B). Similarly, the global age-standardized YLL rate of both overall and smoking attributable MS is expected to decline from 19.75 and 2.22 per 100,000 populations in 2020 to 14.40 and 1.44 per 100,000 populations in 2040, respectively (Figures 7C,D).

## 4 Discussion

MS is a common immune-mediated chronic neurodegenerative disease that damages the central nervous system and significantly impacts the quality of life of patients, resulting in an overwhelming socio-economic burden and loss of labor resources for families, communities, and society as a whole. Unlike young-onset MS, which often presents with a relapsing–remitting course, old-onset MS is more likely to demonstrate a progressive disease course from onset, making it difficult to diagnose and manage (9). The epidemiological patterns and temporal trends of MS among older adults worldwide are of particular interest, given the aging population in many countries. Based on dataset from the GBD study 2019, the current evaluation provides a systematic and up-to-date analysis of the burden of overall and smoking-attributable MS among older adults on a global, regional, and national scale in 2019, and assessed the temporal trends over the past 30 years.

Our findings highlight the significant burden of MS among older adults, a demographic that has been less frequently examined in previous research. While the incidence of MS generally peaks in younger adults, the disease burden and progression in older patients are notable due to the cumulative effects of aging on disease trajectory and treatment response (35). The reduced efficacy of MS therapies in older adults may be attributed to several age-related factors. Physiological changes with aging, such as decreased renal and hepatic function, can alter drug metabolism and clearance (36). Additionally, the immune system undergoes senescence, characterized by diminished immune responses and increased inflammation, which can impact the effectiveness of therapies, which highlights that age-related immune dysfunction can complicate MS treatment and disease management (11). Furthermore, older adults with MS are more likely to experience comorbidities such as cardiovascular disease and diabetes, which can exacerbate MS symptoms and complicate treatment. These comorbidities can contribute to accelerated neurodegeneration and increased disability (37).

There was a substantial disparity in MS burden among the elderly population between regions. Consistent with the previous study that confirmed the positive correlation between age-standardized rate of DALYs for MS for all age groups and the SDI level (14), in 2019, both the absolute number and age-standardized rate of YLD and YLL due to overall and smoking MS tend to increase with the SDI level, suggesting that socioeconomic factors play a role, either as a confounder or mediator, in the distribution variations of the burden of MS (38, 39). High-income North America and Western Europe had the largest burden of overall and smoking-attributable MS among older adults in 2019. Previous investigations show a significant increase in the MS incidence with latitude, with the highest incidence reported in North America and Northern Europe (40–42). The primary environmental factor believed to be responsible for this trend is ultraviolet radiation and vitamin D (43, 44). However, the geographic space is three-dimensional, with altitude being another dimension, and there is a 1.04 time increase in the prevalence of vitamin D insufficiency per 100 m above sea level (45). Furthermore, socioeconomic status has been positively associated with vitamin D deficiency. One potential explanation for this relationship is that individuals of lower socioeconomic status may have greater work- or leisure-related sun exposure (45, 46).

Our results found that on a global level, more than 60% MS-related DALYs among older adults in 2019 came from YLL, suggesting that the total health loss from MS was primarily associated with premature death. Notably, the percentage of DALYs attributed to YLD increased with higher SDI quintiles. This greater relative contribution of YLDs in higher SDI settings is likely due to better access to treatment and improved survival rates (47). As a result, the contribution of YLDs to health loss due to MS is expected to become increasingly significant in global health planning, given the extended life expectancy of MS patients (48). Therefore, the support needs of MS survivors should be integrated into comprehensive MS control planning efforts.

From 1990 to 2019, there was a significant increase in the absolute number of YLD and YLL caused by MS, by 0.99-fold and1.02-fold, respectively. However, the age-standardized YLD and YLL rates tend to trend downward globally in the same period. The increase of absolute number of MS burden can be attributed to improved life expectancy (population aging) and populations growth, while the decreases in age-standardized rate of MS burden were a result of advancements in prevention, diagnosis, and therapies (8, 26, 49, 50). Although the global age-standardized rate of YLD for MS among older adults decreased from 1990 to 2019, a significant increase was observed in many regions, particularly in East Asia. Conversely, the age-standardized YLL rate of MS in East Asia decreased significantly during the same period. Similarly, previous studies of the aforementioned regions have documented increasing trends in MS prevalence and declining mortality of MS (51, 52). Although MS is less common in East Asia, Taiwan had the largest increase in age-standardized rate of incidence and prevalence worldwide. The rising burden of MS in Taiwan were relatively consistent with previous reports (53), and may be associated with the heavy metal exposure (54). Moreover, the proportion of DALYs attributed to YLDs in the overall and smoking-attributable MS cases increased from 1990 to 2019 across all SDI quintiles (barring the high SDI quintile). While global public health efforts have primarily focused on life-saving interventions targeting the leading causes of death, it is crucial to recognize the growing significance of disability as a major contributor to the overall burden of disease and healthcare expenditure (26, 55). Given people with MS living longer and the rapid aging of the population, policymakers must proactively anticipate these changes and adequately allocate resources to healthcare services that can effectively manage the rising prevalence of disabling conditions in older adults with MS (9, 26, 56). Thus, a thorough assessment of the current status, trends, and even future projections regarding the MS burden in the elderly population will shed light on the developing healthcare services scenario. This includes the essential infrastructure and adequately trained personnel required to tackle these emerging challenges.

Smoking reportedly affects MS patients throughout the course of the disease, from the onset of MS to its progression (57, 58), and may compromise the efficacy of disease-modifying drugs like interferon-β (59). The geographic variation in age-standardized mortality rate attributable to smoking can attributed to the difference in smoking prevalence (60). The regions with the highest age-standardized YLD and YLL rates for MS attributable to smoking were High-income North America and Western Europe. Encouragingly, due to worldwide progress in tobacco control, since 1990 the global prevalence of smoking has decreased by 27.5% for males and 37.7% for females (60). As a result, the burden of MS attributable to smoking in the elderly population has also reduced from 1990 to 2019 globally, particularly in the high and high-middle SDI quintiles. Therefore, a considerable proportion of MS burden are preventable by adhering to the guidelines for a healthy lifestyle and well-being that everyone would benefit from.

While sex differences in life expectancy and gendered social determinants of health may partly account for the higher prevalence of MS in females, there are also various sex-specific etiological factors or biological mechanisms underlying the increased female preponderance of MS, including epigenetics, endocrine factors, and modern lifestyle (for example, occupation, cigarette smoking, obesity, birth control and childbirth) (4, 61). Further research should focus on the impact of sex-specific factors on the risk and disease trajectory for MS. Notably, the burden of MS related to smoking is greater in males versus females. This difference is a result of the variation in tobacco consumption between males and females. It is reported that the age-standardized prevalence rate of daily smoking worldwide in 2019 was 32.7% for males and 6.62% for females (60).

The GBD 2016 Multiple Sclerosis Collaborators have outlined the global, regional, and national burden and trend of MS from 1990 to 2016 (3). However, with each new edition of the GBD, data are updated and new methods are employed; therefore, estimates for the entire time series supplant previously reported GBD round estimates (26). In comparison to prior GBD studies, GBD study 2019 has integrated new systematic reviews, cohorts, trials, and case–control studies. Moreover, not only does this current study encompass data for 2017, 2018 and 2019, but it also expands its survey coverage from 195 to 204 countries or territories. Furthermore, two studies have described the distribution and trend of MS burden globally and in China from 1990 to 2019 by using the GBD Study 2019, respectively (14, 51). No study has specifically evaluated the global burden of overall and smoking-attributable MS burden and its secular trend among older adults, however, and the variations by sex and between regions with different levels of socio-economic development. Furthermore, the Bayesian age-period-cohort model was utilized in our study to predict the age-standardized incidence and mortality rates for MS from 2020 to 2040.

Older adults with MS may have different responses to treatments due to age-related physiological changes, necessitating the development of specialized guidelines to enhance management and improve outcomes. Therefore, it is essential to create and implement guidelines specifically tailored for managing MS in older adults, taking into account their unique physiological changes, comorbidities, and the reduced efficacy of disease-modifying therapies. Additionally, educational campaigns should be launched to raise awareness about the impact of smoking on MS and emphasize the importance of early diagnosis and treatment for older adults to mitigate the long-term effects of the disease. Future research should focus on how common comorbid conditions in older adults influence MS management and treatment outcomes. A better understanding of the interplay between MS and comorbidities will enable the development of more integrated and effective management strategies for older patients.

## 5 Limitations

There are some limitations in the current study. Firstly, this study did not evaluate the distribution and trend of disease burden for MS by subtype. According to the clinical course, MS can be defined into four phenotypes: clinically isolated syndrome (CIS), relapsing–remitting MS (RRMS), primary progressive MS (PPMS), and secondary progressive multiple sclerosis (SPMS) (62). Research has shown that PPMS had a higher prevalence in males across all age groups (63), whereas RRMS, which accounts for approximately 80–85% of MS cases (62), predominantly affects females (43). These differences suggest that various MS subtypes have distinct risk factors. By combining the burden of all MS subtypes, we might obscure the changing etiology, despite improving statistical power. Accurately evaluating the burden of each MS form is essential for resource allocation and treatment decisions. Secondly, while we estimated the fraction of MS disease burden attributable to smoking, we did not adequately assess other well-established risk factors. Smoking is a globally recognized issue, with widespread efforts to reduce the number of smokers and minimize passive smoking in public places. By focusing solely on smoking, our study may overlook other significant risk factors that could inform prevention strategies. This limitation means that our findings and interpretations may not fully address the multifaceted nature of MS risk factors. Future research should aim to provide a more comprehensive assessment of all relevant risk factors to enhance prevention efforts. Thirdly, the accuracy and robustness of the GBD estimates depend on the quality and quantity of the original data or publications, potentially introducing bias due to the lack of specific diagnostic biomarkers and evolving diagnostic criteria for MS (64). Inadequate data, especially from underdeveloped regions, may lead to an underestimation of the disease burden and affect the precision of our estimates. This data deficiency introduces uncertainty into the observed trends, as evidenced by the wide uncertainty bounds in the health loss estimates. Consequently, our findings and interpretations might not fully capture the true extent and distribution of MS burden globally. Future research should prioritize the inclusion of more and higher-quality studies to improve the accuracy and reliability of these estimates.

## 6 Conclusion

Our study contributes to a deeper understanding of the MS burden by focusing on older adults and evaluating the impact of smoking. These findings offer a crucial basis for informed public health and healthcare decisions. We highlight the necessity for customized interventions and treatment approaches that address the specific challenges encountered by older MS patients and the ongoing influence of smoking.

## Data Availability

The original contributions presented in the study are included in the article/Supplementary material, further inquiries can be directed to the corresponding authors.

## Footnotes

^1^ http://www.healthdata.org/gbd/about/protocol

^2^ http://ghdx.healthdata.org/gbd-results-tool

^3^ https://seer.cancer.gov/stdpopulations/world.who.html

## Supplementary material

SUPPLEMENTARY FIGURE S1 Percent contribution of YLLs and YLDs in DALYs of overall **(A)** and smoking-attributable **(B)** multiple sclerosis among older adults aged 65–89 years at global level and in 5 SDI quintiles, 1990–2019. YLL, years of life lost; YLD, years of life lived with disability; DALYs, disability-adjusted life years. DALYs are the sum of YLLs and YLDs.

SUPPLEMENTARY FIGURE S2 Estimated annual percentage change (EAPC) of the age-standardized rates of YLD and YLL for overall and smoking-attributable multiple sclerosis among older adults aged 65–89 years from 1990 to 2019 versus the 2019 SDI level. **(A)** EAPC of age-standardized YLD rate for MS versus the 2019 SDI level; **(B)** EAPC of age-standardized YLD rate for MS attributable to smoking versus the 2019 SDI level; **(C)** EAPC of age-standardized YLL rate for MS versus the 2019 SDI level; **(D)** EAPC of age-standardized YLL rate for MS attributable to smoking versus the 2019 SDI level. YLD, years of life lived with disability; YLL, years of life lost; SDI, socio-demographic index.

SUPPLEMENTARY FIGURE S3 Temporal patterns of YLD caused by multiple sclerosis attributable to smoking in older adults aged 65–89 years worldwide and in 5 SDI quintiles, 1990–2019. **(A)** The number of YLD; **(B)** Age-standardized YLD rate (per 100,000). YLD, years of life lived with disability; SDI, socio-demographic index.

SUPPLEMENTARY FIGURE S4 Temporal patterns of YLL caused by multiple sclerosis attributable to smoking in older adults aged 65–89 years worldwide and in 5 SDI quintiles, 1990–2019. **(A)** The number of YLL; **(B)** Age-standardized YLL rate (per 100,000). YLL, years of life lost; SDI, socio-demographic index.

SUPPLEMENTARY FIGURE S5 Age-specific rates of MS burden by age group. **(A)** Age-specific rates of YLD and YLL caused by multiple sclerosis in 1990 (Dash line) and 2019 (Solid line), respectively, are shown by age. **(B)** The trend of age-specific rate of DALYs due to multiple sclerosis by age from 1990 to 2019 for male. **(C)** The trend of age-specific rate of DALYs due to multiple sclerosis by age from 1990 to 2019 for female. YLD, years of life lived with disability; YLL, years of life lost; DALYs, disability-adjusted life years.

SUPPLEMENTARY FIGURE S6 Age-specific rate of YLD for multiple sclerosis by age group across the globe, 5 SDI quintiles, and 21 GBD regions, 1990–2019. YLD, years of life lived with disability; SDI, socio-demographic index.

SUPPLEMENTARY FIGURE S7 Age-specific rate of YLL for multiple sclerosis by age group across the globe, 5 SDI quintiles, and 21 GBD regions, 1990–2019. YLL, years of life lost; SDI, socio-demographic index.

SUPPLEMENTARY FIGURE S8 Age-specific rate of YLD for smoking-related multiple sclerosis by age group across the globe, 5 SDI quintiles, and 21 GBD regions, 1990–2019. YLD, years of life lived with disability; SDI, socio-demographic index.

SUPPLEMENTARY FIGURE S9 Age-specific rate of YLL for smoking-related multiple sclerosis by age group across the globe, 5 SDI quintiles, and 21 GBD regions, 1990–2019. YLL, years of life lost; SDI, socio-demographic index.

SUPPLEMENTARY FIGURE S10 The fractions of YLD caused by multiple sclerosis attributable to smoking are illustrated by age group across the globe, 5 SDI quintiles, and 21 GBD regions, 1990–2019. YLD, years of life lived with disability; SDI, socio-demographic index.

SUPPLEMENTARY FIGURE S11 The fractions of YLL caused by multiple sclerosis attributable to smoking are illustrated by age group across the globe, 5 SDI quintiles, and 21 GBD regions, 1990–2019. YLL, years of life lost; SDI, socio-demographic index.
