# Supplementary figures and images for: The overall and smoking-attributable burden of multiple sclerosis among older adults aged 65–89 years from 1990 to 2019 and predictions to 2040
Source: Front Med (Lausanne). 2024 Aug 22;11:1430741. doi: 10.3389/fmed.2024.1430741 (PMC11374621; doi:10.3389/fmed.2024.1430741)

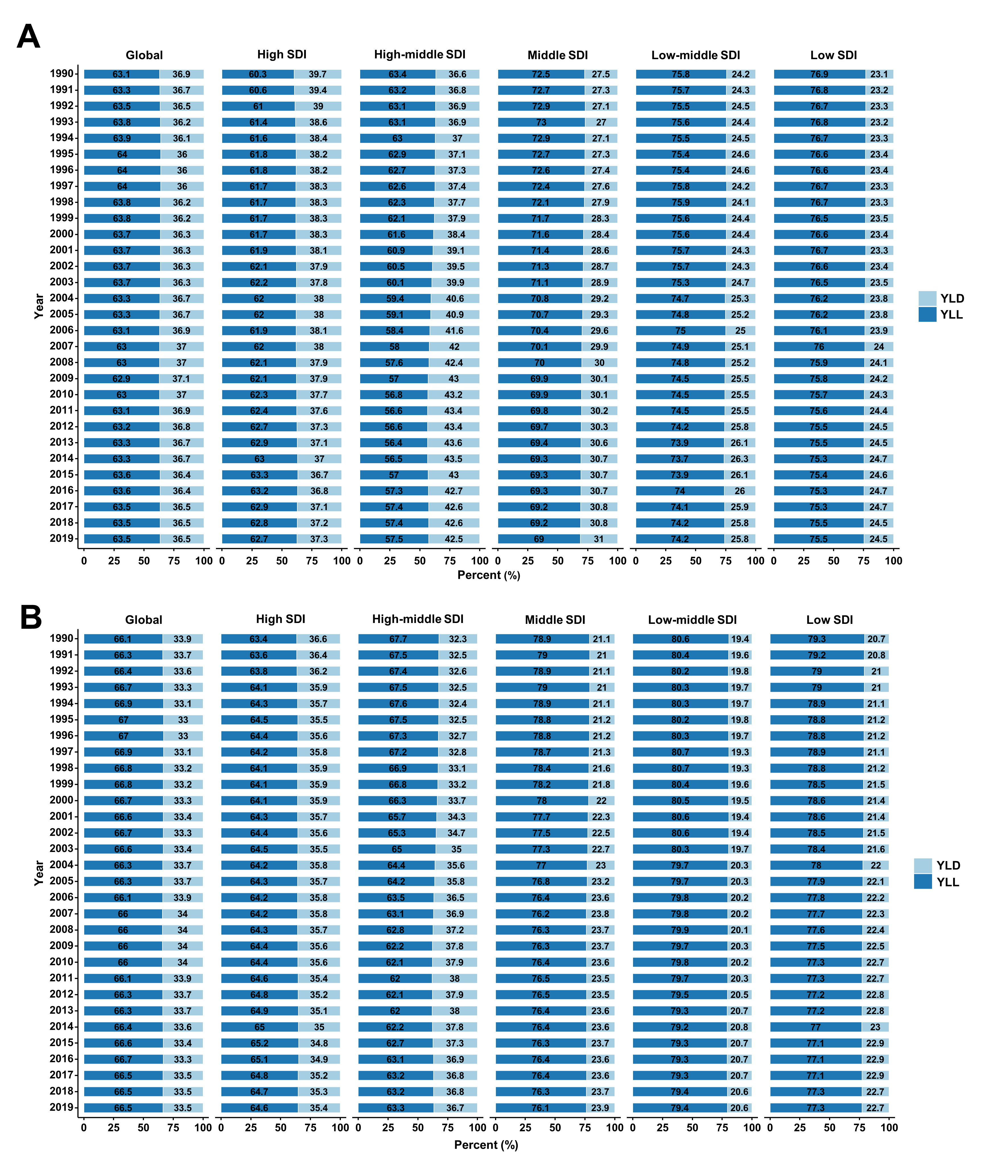

Supplement: SUPPLEMENTARY FIGURE S1 — Percent contribution of YLLs and YLDs in DALYs of overall (A) and smoking-attributable (B) multiple sclerosis among older adults aged 65–89 years at global level and in 5 SDI quintiles, 1990–2019. YLL, years of life lost; YLD, years of life lived with disability; DALYs, disability-adjusted life years. DALYs are the sum of YLLs and YLDs. [file Image_1.TIF]

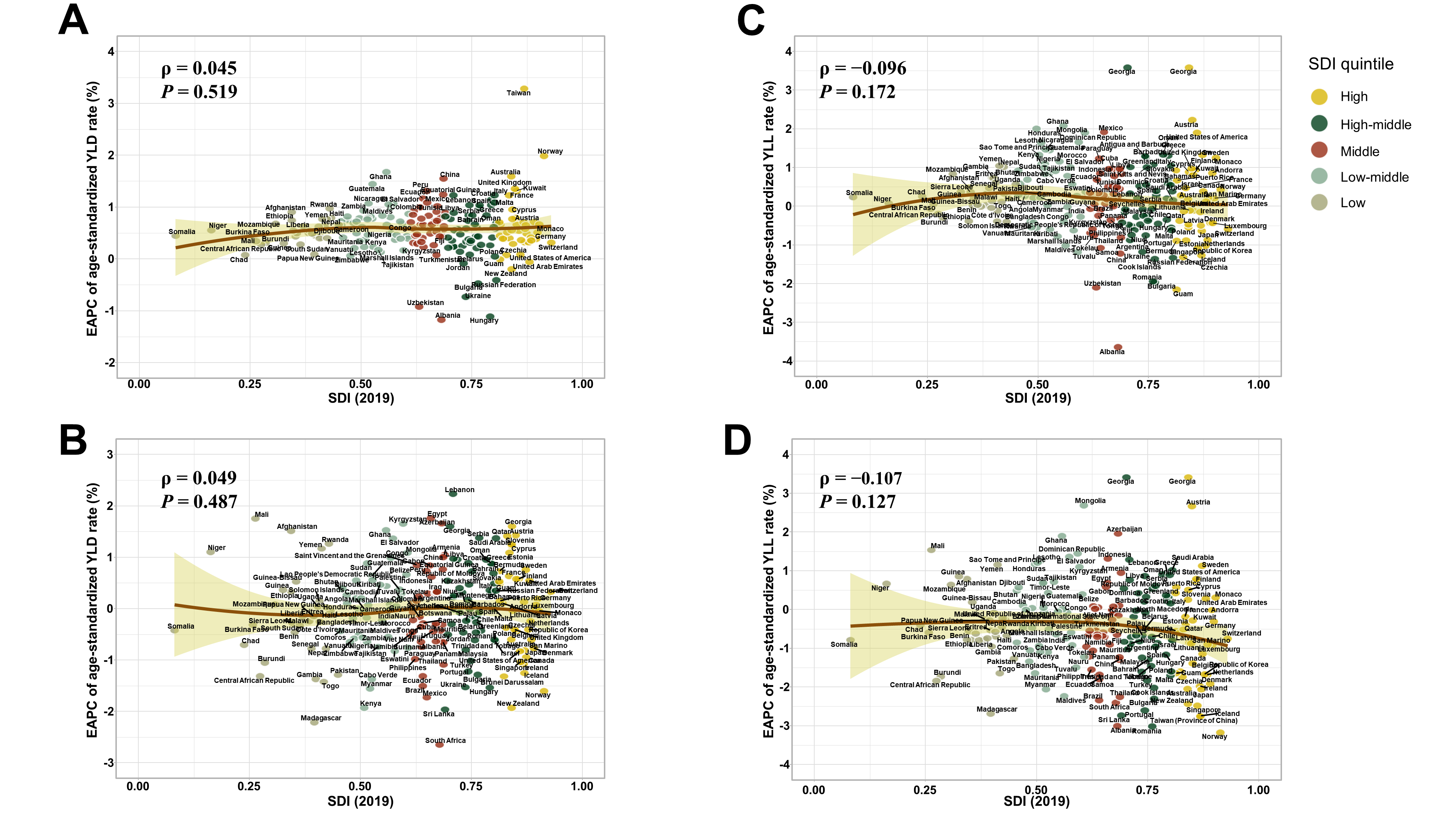

Supplement: SUPPLEMENTARY FIGURE S2 — Estimated annual percentage change (EAPC) of the age-standardized rates of YLD and YLL for overall and smoking-attributable multiple sclerosis among older adults aged 65–89 years from 1990 to 2019 versus the 2019 SDI level. (A) EAPC of age-standardized YLD rate for MS versus the 2019 SDI level; (B) EAPC of age-standardized YLD rate for MS attributable to smoking versus the 2019 SDI level; (C) EAPC of age-standardized YLL rate for MS versus the 2019 SDI level; (D) EAPC of age-standardized YLL rate for MS attributable to smoking versus the 2019 SDI level. YLD, years of life lived with disability; YLL, years of life lost; SDI, socio-demographic index. [file Image_2.TIF]

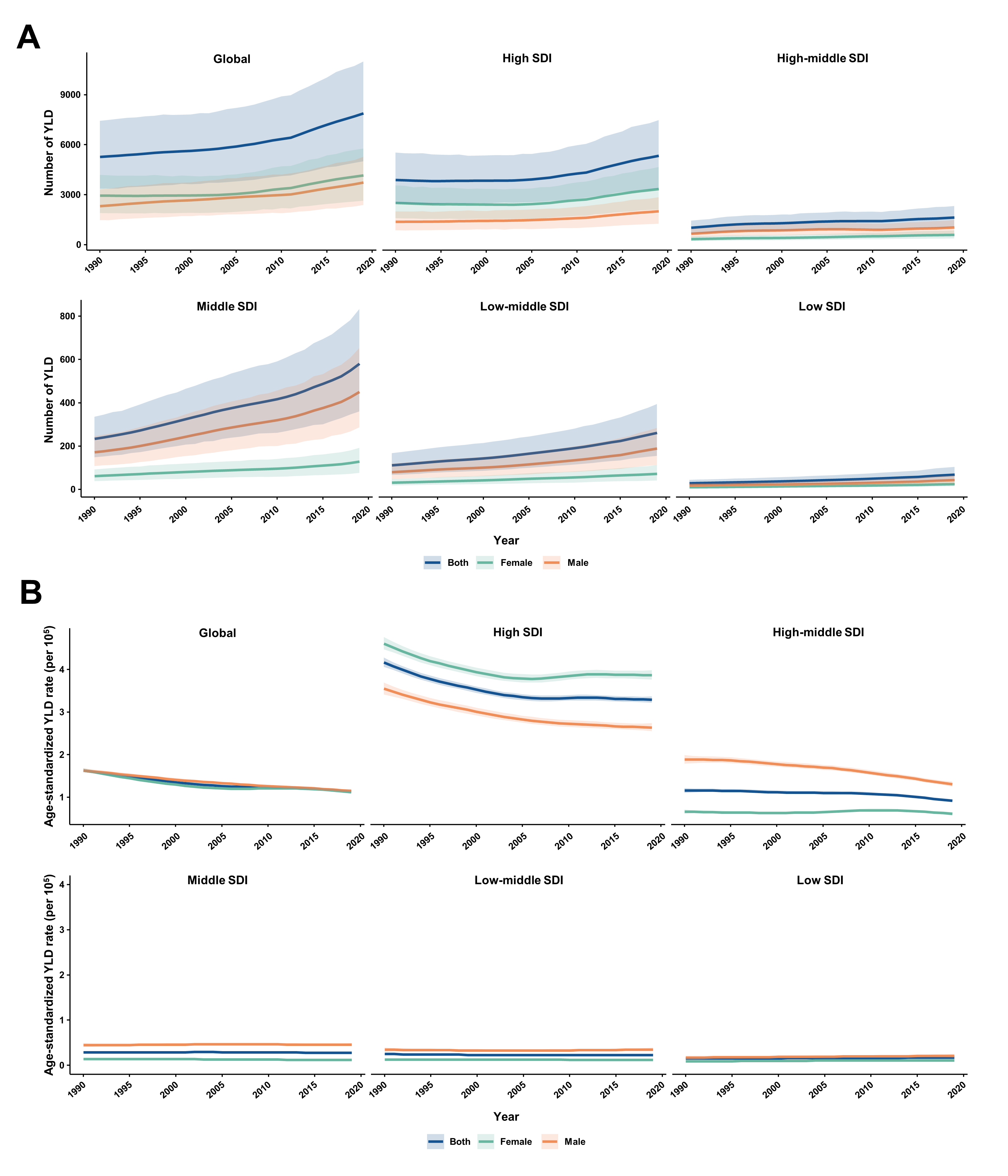

Supplement: SUPPLEMENTARY FIGURE S3 — Temporal patterns of YLD caused by multiple sclerosis attributable to smoking in older adults aged 65–89 years worldwide and in 5 SDI quintiles, 1990–2019. (A) The number of YLD; (B) Age-standardized YLD rate (per 100,000). YLD, years of life lived with disability; SDI, socio-demographic index. [file Image_3.TIF]

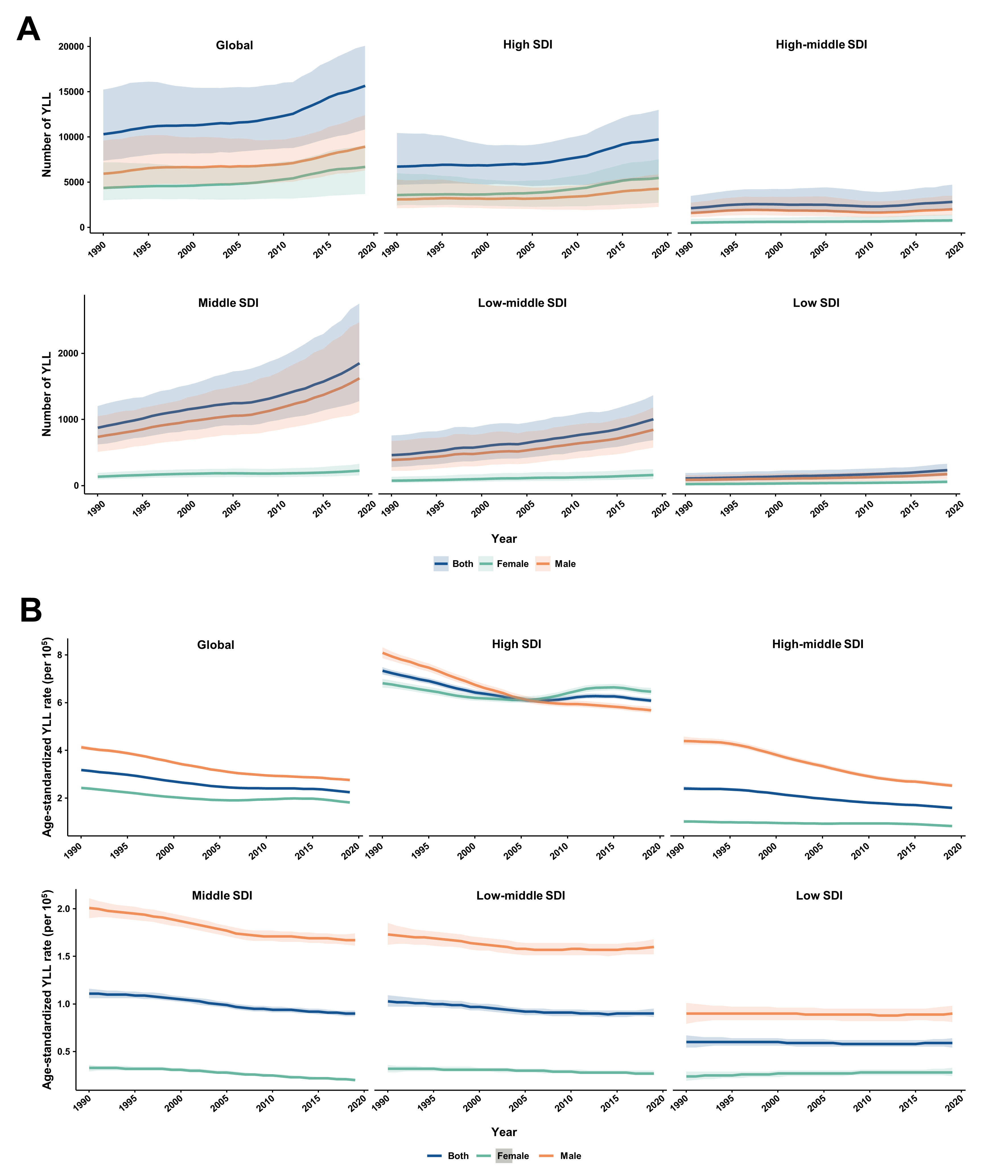

Supplement: SUPPLEMENTARY FIGURE S4 — Temporal patterns of YLL caused by multiple sclerosis attributable to smoking in older adults aged 65–89 years worldwide and in 5 SDI quintiles, 1990–2019. (A) The number of YLL; (B) Age-standardized YLL rate (per 100,000). YLL, years of life lost; SDI, socio-demographic index. [file Image_4.TIF]

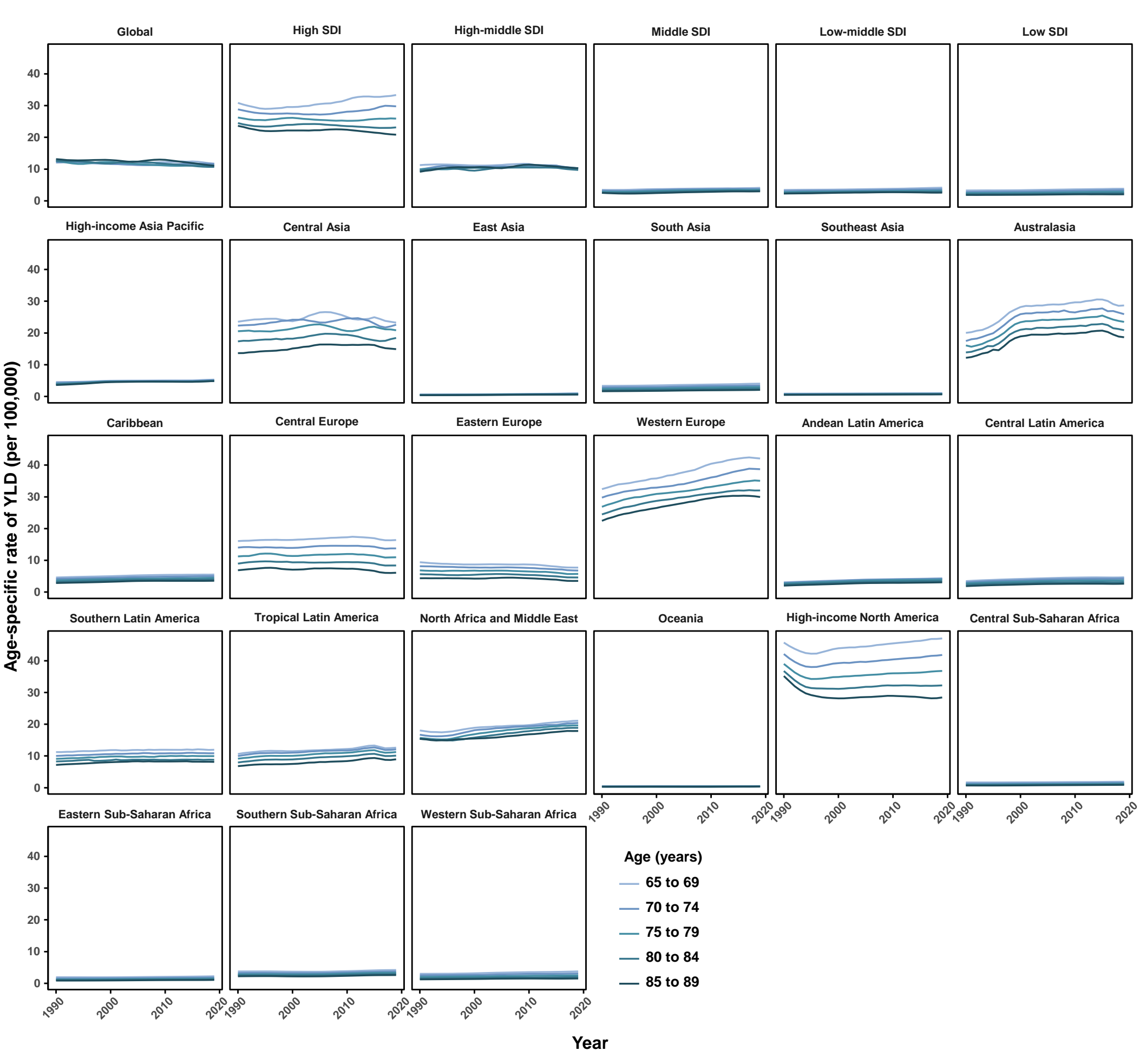

Supplement: SUPPLEMENTARY FIGURE S6 — Age-specific rate of YLD for multiple sclerosis by age group across the globe, 5 SDI quintiles, and 21 GBD regions, 1990–2019. YLD, years of life lived with disability; SDI, socio-demographic index. [file Image_6.PDF]

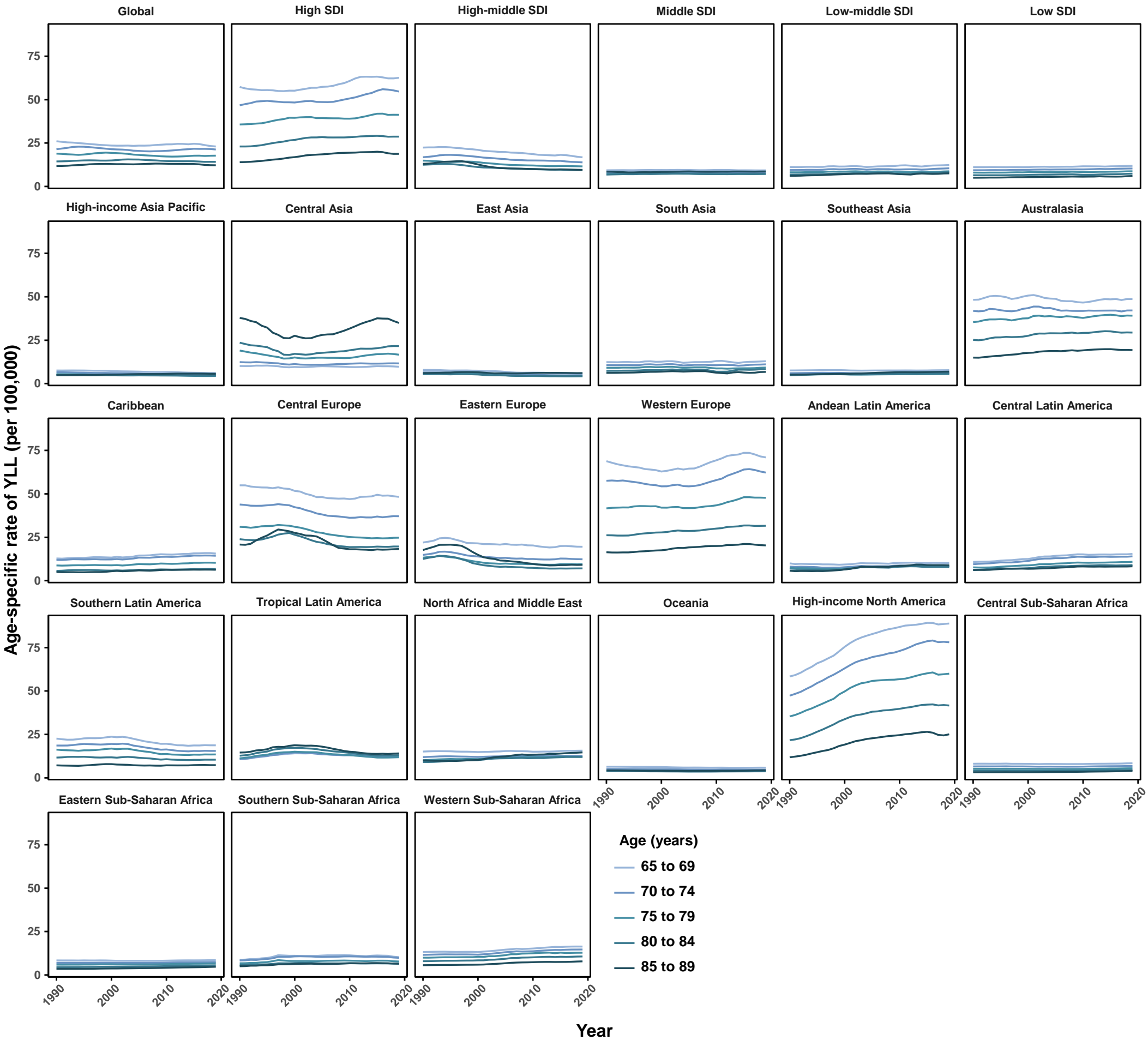

Supplement: SUPPLEMENTARY FIGURE S7 — Age-specific rate of YLL for multiple sclerosis by age group across the globe, 5 SDI quintiles, and 21 GBD regions, 1990–2019. YLL, years of life lost; SDI, socio-demographic index. [file Image_7.PDF]

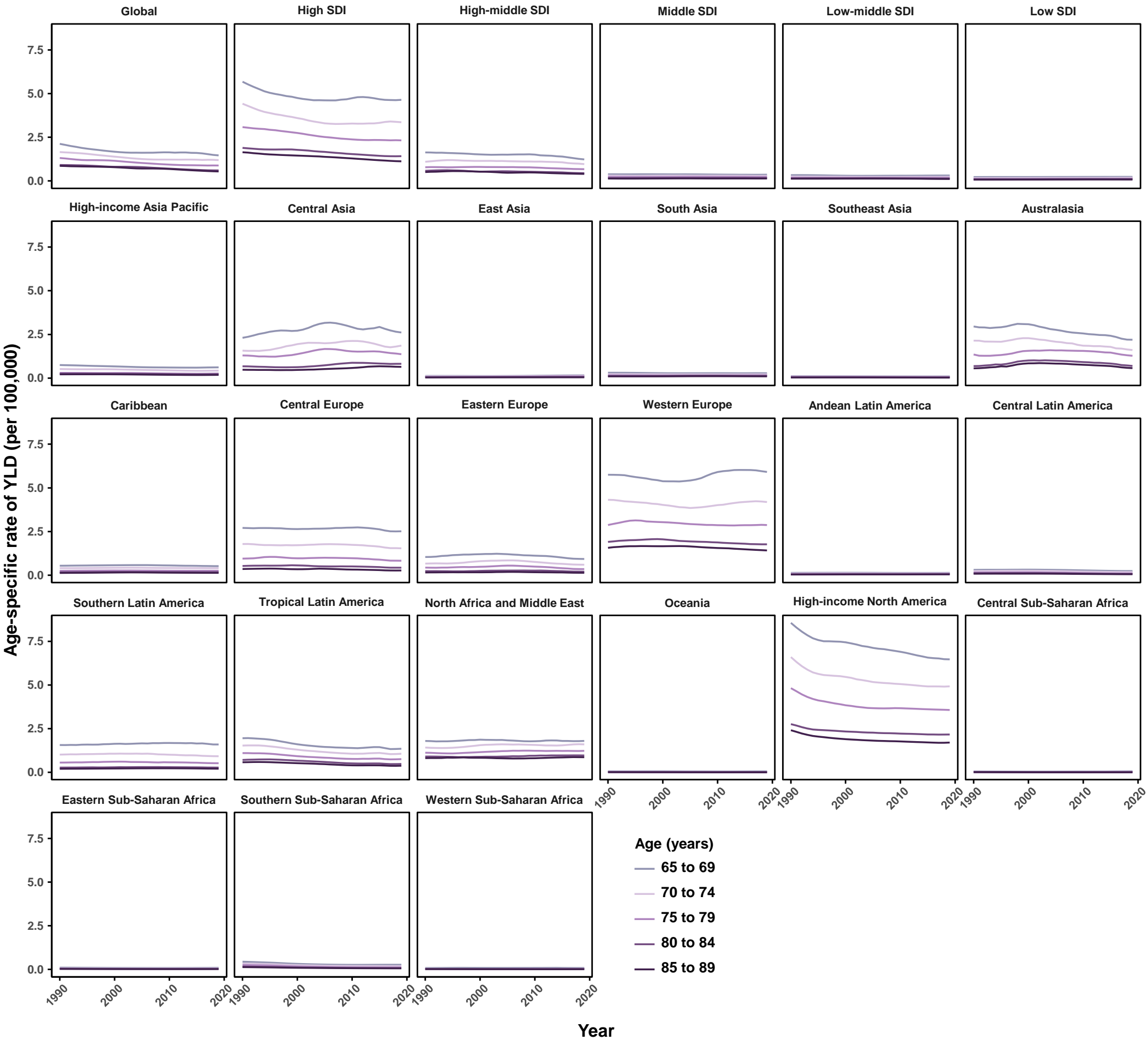

Supplement: SUPPLEMENTARY FIGURE S8 — Age-specific rate of YLD for smoking-related multiple sclerosis by age group across the globe, 5 SDI quintiles, and 21 GBD regions, 1990–2019. YLD, years of life lived with disability; SDI, socio-demographic index. [file Image_8.PDF]

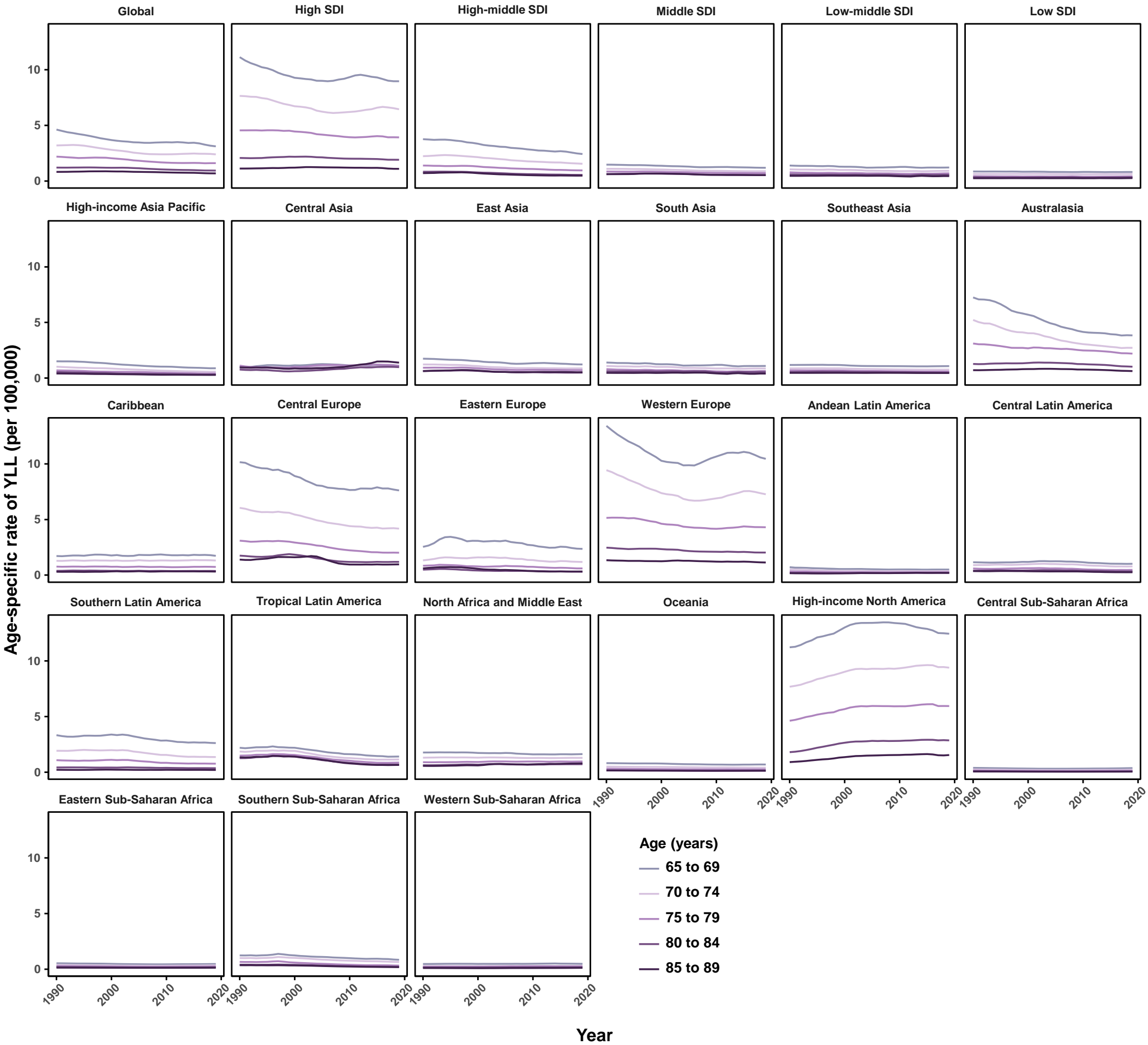

Supplement: SUPPLEMENTARY FIGURE S9 — Age-specific rate of YLL for smoking-related multiple sclerosis by age group across the globe, 5 SDI quintiles, and 21 GBD regions, 1990–2019. YLL, years of life lost; SDI, socio-demographic index. [file Image_9.PDF]

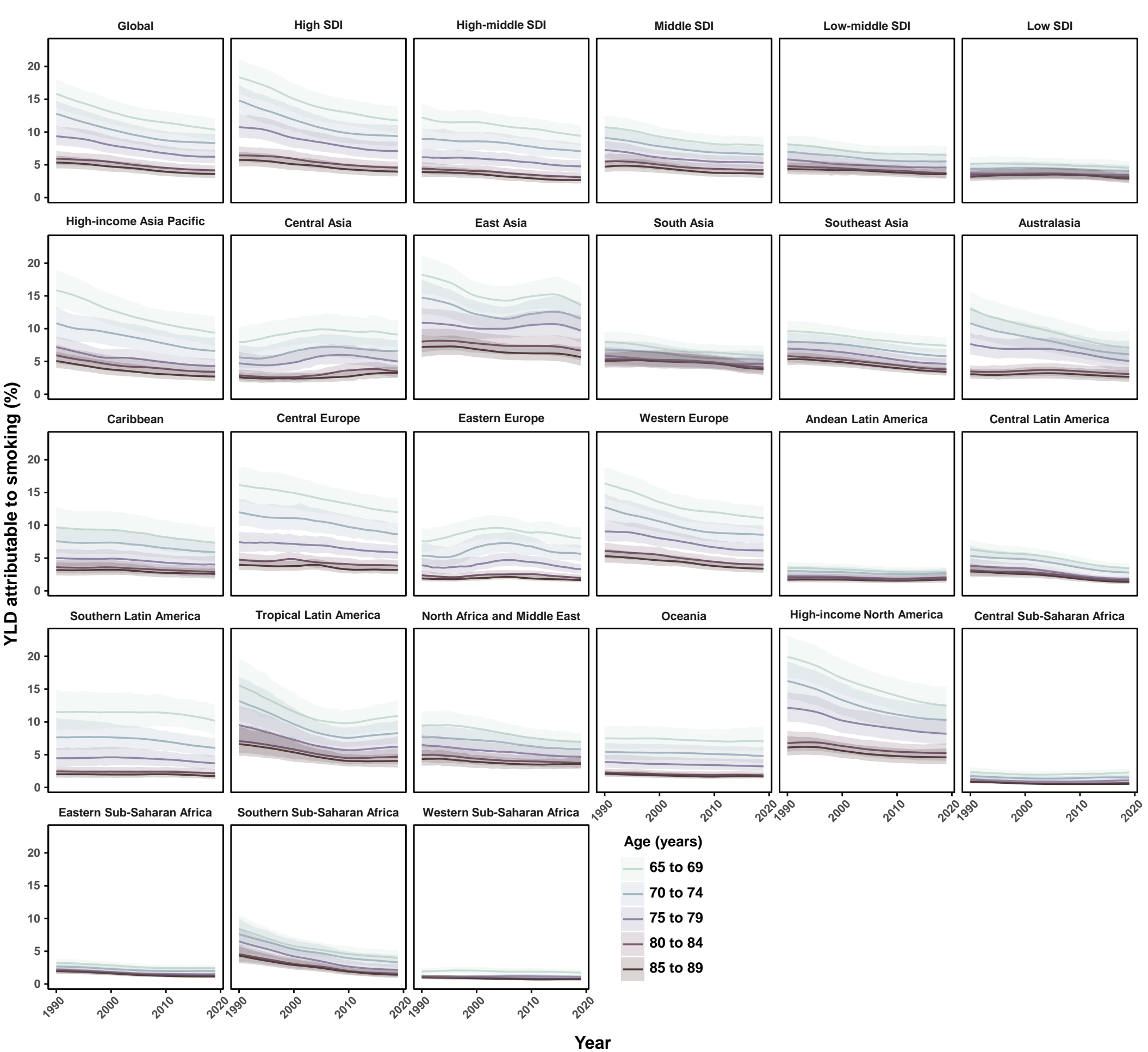

Supplement: SUPPLEMENTARY FIGURE S10 — The fractions of YLD caused by multiple sclerosis attributable to smoking are illustrated by age group across the globe, 5 SDI quintiles, and 21 GBD regions, 1990–2019. YLD, years of life lived with disability; SDI, socio-demographic index. [file Image_10.PDF]

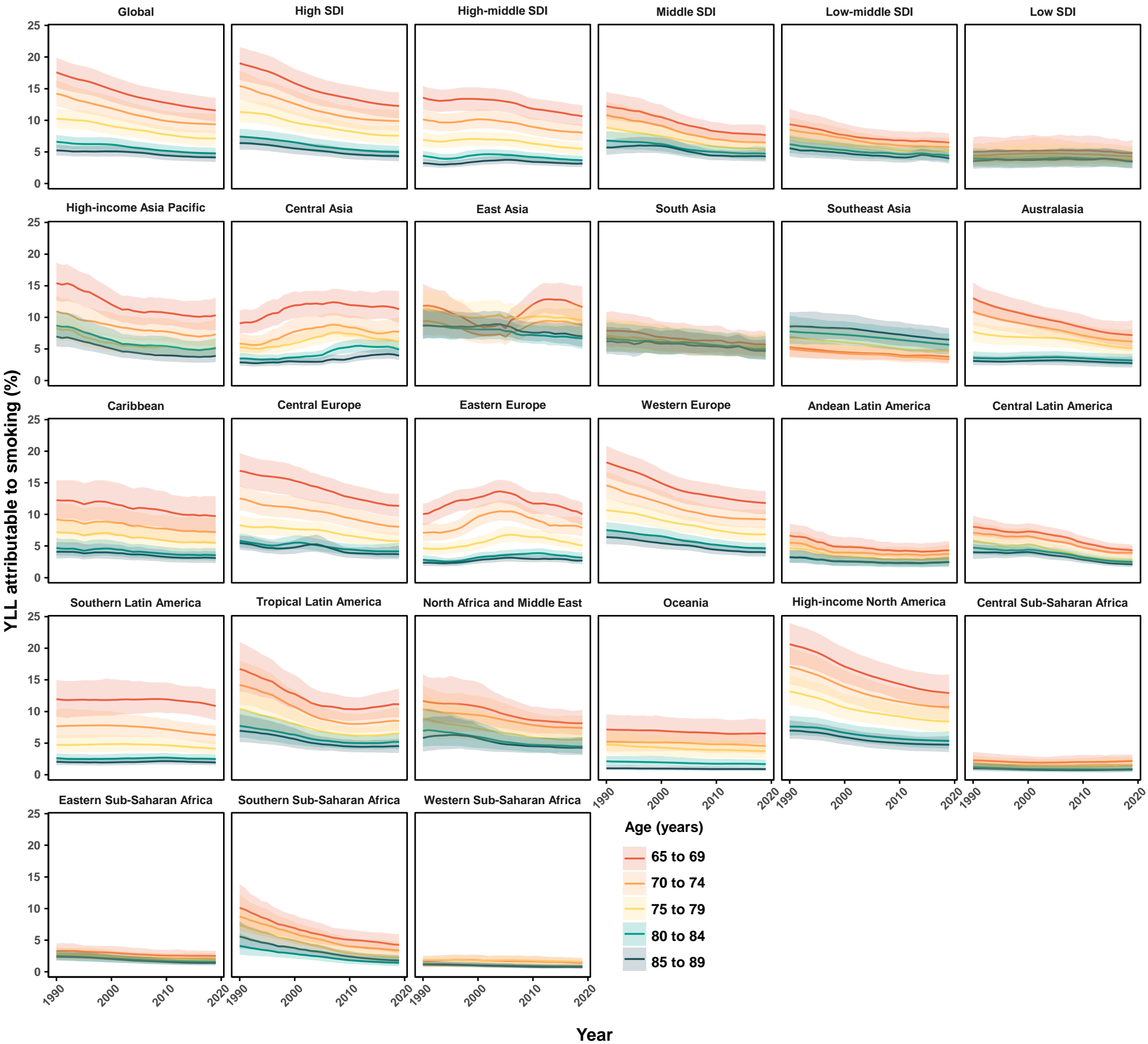

Supplement: SUPPLEMENTARY FIGURE S11 — The fractions of YLL caused by multiple sclerosis attributable to smoking are illustrated by age group across the globe, 5 SDI quintiles, and 21 GBD regions, 1990–2019. YLL, years of life lost; SDI, socio-demographic index. [file Image_11.PDF]
